# Overactivation of Cdc42 GTPase Impairs the Cytotoxic Function of NK Cells From Old Individuals Towards Senescent Fibroblasts

**DOI:** 10.1111/acel.70398

**Published:** 2026-02-08

**Authors:** Albert Kallon Koroma, Karmveer Singh, Yongfang Wang, Linda Krug, Philipp Haas, Meinhard Wlaschek, Vadim Sakk, Tanja Schuster, Daniel Fürst, Hubert Schrezenmeier, Rashmi Priyadharshini Dheenadayalan, Stephan Stilgenbauer, Lutz Walter, Hartmut Geiger, Karin Scharffetter‐Kochanek, Pallab Maity

**Affiliations:** ^1^ Department of Dermatology and Allergic Diseases Ulm University Ulm Germany; ^2^ Aging Research Center (ARC) Ulm University Ulm Germany; ^3^ Institute of Molecular Medicine Ulm University Ulm Germany; ^4^ Department of Transplantation Immunology, Institute for Clinical Transfusion Medicine and Immunogenetics Ulm University, German Red Cross Blood Transfusion Service Ulm Germany; ^5^ Institute of Transfusion Medicine Ulm University Ulm Germany; ^6^ Internal Medicine III Ulm University Ulm Germany; ^7^ Comprehensive Cancer Center Ulm Ulm University Ulm Germany; ^8^ Primate Genetics Laboratory, German Primate Centre Leibniz Institute for Primate Research Göttingen Germany

**Keywords:** aging, CASIN, Cdc42, granzyme B, malignant lymphoma cells, NK cell, perforin, senescent fibroblasts

## Abstract

Senescent fibroblasts accumulate in the connective tissue of all organs and promote organ aging and aging‐related diseases. The underlying mechanisms for the accumulation of senescent fibroblasts are poorly understood. Natural killer (NK) cells of innate immunity play a critical role in the removal of tissue resident senescent cells. We here show that NK cells from old adults and old mice fail to efficiently remove senescent fibroblasts. This is due to severely reduced perforin and granzyme B release from aged NK cells where perforin is responsible for inducing holes in the membrane of senescent fibroblasts through which granzyme B enters enforcing cell death of senescent fibroblasts. We demonstrate elevated activation of the small Cdc42 Rho GTPase in aged NK cells to be responsible for the disruption of the microtubular organization which is essential for the proper release of perforin and granzyme B and for energy homeostasis. Attenuation of the elevated activity of Cdc42 in aged human NK cells with CASIN, a small molecule Cdc42 inhibitor, rebalances Cdc42 activity to a young level. Rebalancing of Cdc42 restores proper perforin and granzyme B release and attenuates reduced ATP levels in aged NK cells resulting in an attenuated “youthful” cytotoxicity of aged NK cells against senescent cells. Collectively, we identified a previously unreported molecular mechanism underlying functional impairment of NK cells from older adults. In perspective, our data hold promise to develop novel strategies against age‐related disorders driven by tissue‐resident senescent fibroblasts.

## Graphical abstract

Unrestrained Cdc42 activity causes failure of cytotoxic function of old NK cells. This is associated with disruption of microtubular network and impaired mitochondrial ATP resulting in reduced conjugation and impaired degranulation of lytic vesicles. Pharmacological inhibition of Cdc42 by CASIN attenuates all these and in part rejuvenates the cytotoxicity.

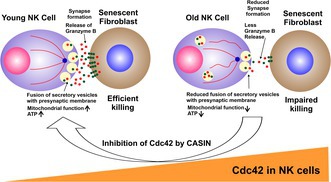

## 1 Introduction

Immune aging involves the gradual decline of both the innate and the adaptive immune system (Camous et al. 2012; Ma and Fang 2013; Crooke et al. 2019). Natural killer (NK) cells belong to the innate branch of the immune system. There is only scant information on the mechanisms of aging of NK cells (Hazeldine et al. 2012).

NK cells counteract, among others, infections and cancer development (Chen et al. 2024). Their role in the control of tissue homeostasis and by the elimination of senescent cells in aged tissue is an emerging concept which, in the long perspective, may be suitable to be exploited for prevention of aging and aging‐related diseases. Cytotoxicity is a highly coordinated multistep process wherein NK cells have the unique capacity to produce and safely transport cytotoxic proteins. These cytotoxic proteins are stored in proteolytic granules and released upon NK cell activation after contact with their target cells. After recognition of target cells, perforin and granzyme B stored in vesicles are transported through microtubules and released at the immune synapse between NK cells and target cells. Perforin punches large pores into the membranes of target cells allowing granzyme B to diffuse into the cytoplasm of target cells where it enforces their apoptosis (Topham and Hewitt 2009; Voskoboinik et al. 2015). NK cells kill target cells by shifting the balance between activating and inhibiting cell surface receptors towards activating receptors (Dorshkind et al. 1985; Lanier 2005; Parham 2005; Sun and Lanier 2011; Wang et al. 2015). Immunostaining of NK cells from old mice revealed a diffuse staining of perforin which implied a reduced ability of these NK cells to form a focused cytotoxic immune synapse (Hazeldine et al. 2012; Ovadya and Krizhanovsky 2014). However, neither the underlying mechanisms of a disturbed immune synapse nor its consequences in NK cell aging have been addressed in detail so far.

Fibroblasts are the principal cellular component of the connective tissue and play an essential role in proper organ homeostasis and regeneration. In contrast, senescent fibroblasts enforce organ aging and contribute to morbidity (Wlaschek et al. 2021). When fibroblasts are exposed to either acute or chronic stress, they undergo cellular senescence. Cellular senescence is defined as the irreversible arrest of cell proliferation, resistance to apoptosis, and enhanced release of pro‐inflammatory substances (Ovadya and Krizhanovsky 2014; Kirkland and Tchkonia 2017; Hernandez‐Segura et al. 2018; Calcinotto et al. 2019), referred to as *S*enescence *A*ssociated *S*ecretory *P*henotype (SASP). The SASP allows senescent fibroblasts to maintain their senescent state and to spread senescence in a paracrine manner to the nearby healthy fibroblasts and other cells within the tissue, organs, and the organism. The SASP contains enhanced concentrations of pro‐inflammatory cytokines, chemokines, and proteases, but almost completely lacks growth factors (Bohm et al. 2025). The persisting SASP suppresses endogenous stem cell niches (Maity et al. 2021) and thereby contributes to age‐related diseases. These pathological conditions include impaired wound healing, suppressed regeneration (Jiang et al. 2018; Maity et al. 2021), osteoarthritis, arteriosclerosis, diabetes, and neurodegenerative disorders (Ovadya and Krizhanovsky 2014; Jeon et al. 2017). Senescent fibroblasts gradually accumulate in the skin and other tissues during aging (Ressler et al. 2006). This, in part, is due to the ability of senescent fibroblasts to evade NK cell cytotoxicity by the up‐regulation of HLA‐E on their surface, which interacts with the inhibitory receptor NKG2A expressed by NK cells to suppress cytotoxic removal of senescent fibroblasts (Pereira et al. 2019). Therefore, the elimination of senescent cells, and in particular senescent fibroblasts, by NK cells is of major importance to maintain and restore tissue and organ regeneration and homeostasis.

Previously, elevated activity of cell division protein 42 (Cdc42), a member of the Rho GTPase family, was reported to cause aging of hematopoietic stem cells (HSC) (Florian and Geiger 2010). Cdc42 plays an important role in the regulation of tubulin and actin organization essential for the maintenance of HSC polarity (Sinha and Yang 2008; Florian and Geiger 2010). Cdc42 oscillates between an active GTP‐bound and an inactive GDP‐bound state. Active Cdc42 regulates a variety of important cellular processes, including the organization of the microtubular cytoskeleton, cell polarity, granule transport, and the focused release of cytotoxic content into a synaptic cleft. Given that HSC are precursors of NK cells, we explored whether NK cells from older adults become inefficient in killing senescent fibroblasts and—if so—whether aged NK cells show disturbances of NK cell polarity. Previously unreported, we here found an unrestrained activation of Cdc42 in aged NK cells that impaired the ability of aged human and murine NK cells to remove senescent fibroblasts. CASIN, a small molecule Cdc42 inhibitor, rebalances Cdc42 activity to a young level, restores a proper perforin and granzyme B release, and attenuates reduced ATP levels and mitochondrial dysfunction in aged NK cells to result in a youthful cytotoxic activity of aged NK cells against senescent cells. Hence, we uncovered a strategy to re‐empower the cytotoxic potency of aged NK cells. In the long term, this holds promise for prevention and therapy of tissue and organ decline in aging and aging‐related diseases.

## 2 Results

### 2.1 Aged NK Cells Show Reduced Cytotoxicity Towards Senescent Fibroblasts

**FIGURE 1:**
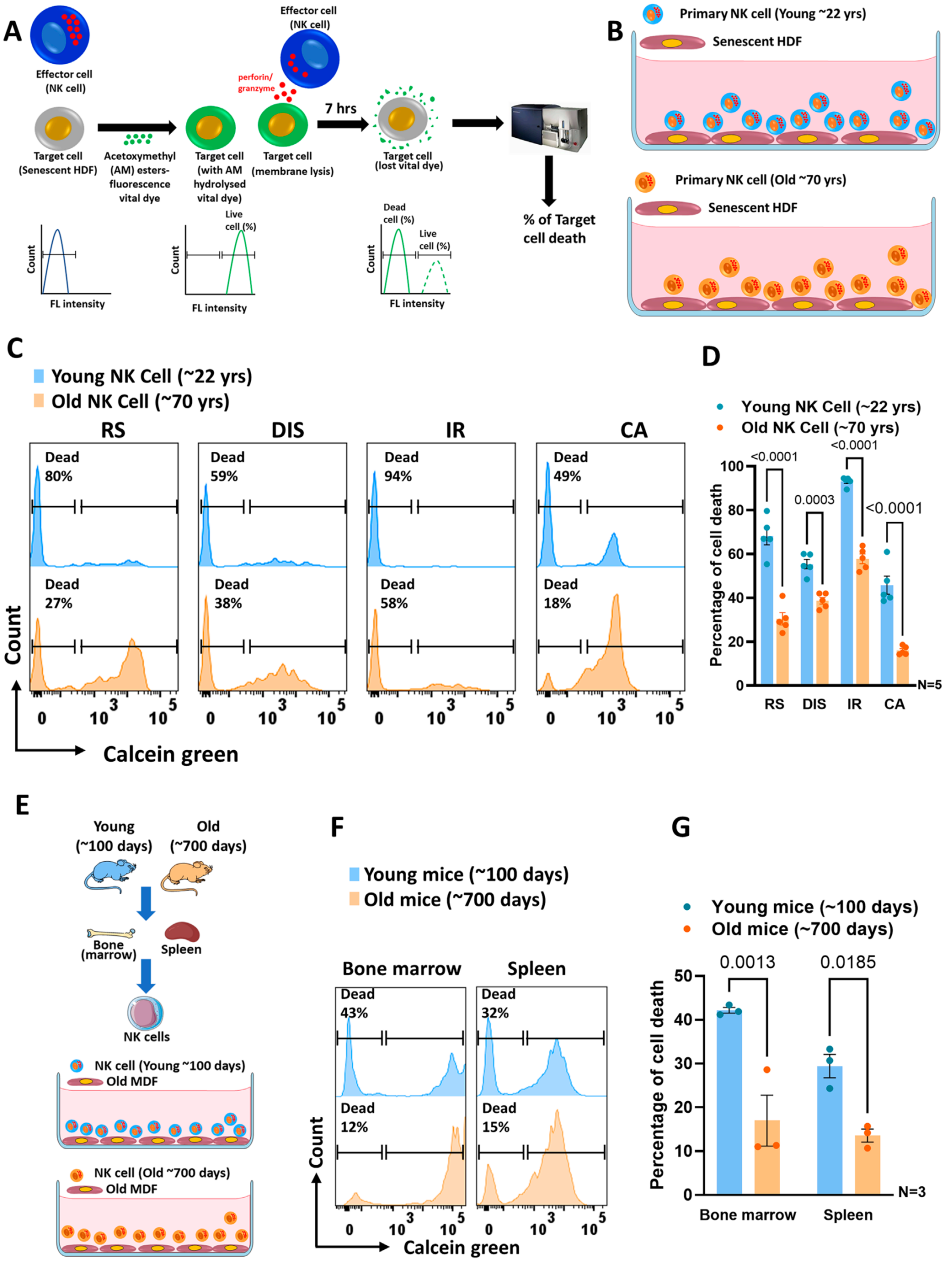
Natural killer cells from old adults reveal reduced cytotoxicity towards senescent fibroblasts. (A) Graphical illustration of the NK cell mediated target cell cytotoxicity assay. Target cells were first stained with calcein acetomethoxymethyl (AM), a vital fluorescent dye. Calcein AM is a non‐fluorescent compound that pass the intact cell membrane into the cytoplasm. Hydrolysis of calcein AM by intracellular esterases in live cells generates calcein, a hydrophilic, intensely fluorescent molecule which reliably stays in the cytoplasm. The stained target cells were next co‐cultured with NK cells isolated from young or old human or mice. NK cells exert their cytotoxicity towards target cells through the release of perforin and granzyme B. Upon lysis of target cells, the calcein dye is released and the loss of the dye is measured as a shift in fluorescence intensity by flow cytometry. Dead cells will appear to the left of the histogram, while alive cells on the right side. The percentage of dead cells can then simply be calculated and presented. (B) Graphical scheme depicts the experimental groups: Co‐cultures of NK cells from young adults with senescent human dermal fibroblasts in the top row and NK cells from old adults with senescent HDF in the bottom row. (C) Histogram (bi‐exponential scale) showing cytotoxicity of NK cells from young and old adults on different senescent HDF. RS, replicative senescent HDF, DIS, doxorubicin induced senescent HDF, IR, ionizing radiation induced senescence, CA, chronologically aged HDF (~75 years). The peak in the left part of the histogram showing the dead cell population and the percentage of dead cells. (D) The graph depicts the percentage of senescent HDF death (*y*‐axis) by NK cells isolated from young and old human adult. Data were represented as mean (Percentage of senescent fibroblast death) ± SEM. *N* = 5. Two tailed Student's *t*‐test was used to assess the significance between young and old groups for each of senescence model. (E) Illustration of the experimental design showing cytotoxic activity of NK cells derived from bone marrow and spleen of young and old mice against aged murine dermal fibroblasts (MDF). (F) Histogram depicting cytotoxicity of NK cells from young and old mice on old MDF. The peak in the left part of the histogram showing the dead cell population and the percentage of dead cells. (G) The graph depicts the percentage of senescent MDF death (*y*‐axis) by NK cells isolated from young and old mice. Data were represented as mean (Percentage of senescent fibroblast death) ± SEM. *N* = 3. Each mouse NK cell sample used in the cytotoxicity assay was the pool of NK cells isolated from three different mice. One‐way ANOVA, followed by Bonferroni multiple comparison test was used to find the significance among the groups.

To test the hypothesis that NK cells undergo, similar to other immune cells, an age‐related decline in their function, we first assessed whether NK cells from older human adults (average age 70 years) and aged mice (average age 700 days) exhibit reduced cytotoxicity against senescent human and murine dermal fibroblasts, in comparison to NK cells from younger human adults (average age 21 years) and young mice (average age 100 days). A purity of > 96% of NK cells (CD56^+^, CD3^−^) irrespective of the donor age was obtained after immunomagnetic negative selection (Figure S1A,B). Their cytotoxic activity was tested against four different types of senescent HDFs: replicative senescent HDFs, doxorubicin induced senescent HDFs, ionizing radiation induced senescent HDFs and aged HDFs isolated from old individuals (average age 75 years old) (Table S1). Senescence of human dermal fibroblasts (HDF) and murine dermal fibroblasts (MDF) was confirmed by the established senescence markers (Ogrodnik et al. 2024) increase of the *S*enescence‐*A*ssociated‐β‐*G*alactosidase (SA‐β‐Gal), and expression of the cyclin kinase inhibitors p16INK4A and p21 (Figure S1C–R). The percentage of cell death of senescent cells was assessed by flow cytometry (Figure 1A–D).

NK cells from older individuals exhibited reduced cytotoxicity towards all four types of senescent HDFs in comparison to NK cells from younger donors (Figure 1B–D). We further observed that NK cells from young adults bounded efficiently to senescent HDFs to form tight and lasting connections, while after killing proceeding to the next target senescent HDF (Video S1). By contrast, NK cells from old individuals could not form such lasting interactions and moved slower to new targets, resulting in impaired killing (Video S2). Next, we investigated whether the observed decline of aged NK cell cytotoxicity was unique towards senescent fibroblasts or did also affect removal of other known targets of NK cells, such as malignant cells. For this purpose, the cytotoxic activity of NK cells from both young and old adults was assessed against a range of primary chronic lymphocytic leukemia (CLL) cells with varying CLL mutation profiles (Table S2), as well as against the chronic myelogenous leukemia cell line K562, chronic lymphocytic leukemia cell lines such as HG3, OSUCLL and the Mantle cell lymphoma cell line JVM2.

We observed a significant reduction in the cytotoxicity of NK cells isolated from old individuals towards all the studied malignant target cells in comparison to the activity of NK cells from young adults (Figure S2A–D). A similar decline in cytotoxic function was also observed for NK cells from old mice. NK cells isolated from the bone marrow and spleen of old mice showed reduced cytotoxicity towards old murine dermal fibroblasts (MDF) (Figure 1E–G) and YAC‐1, a murine lymphoma cell line (Figure S3A,B). All in all, there is an age‐related decline in the cytotoxic NK‐cell function of human and murine NK cells, regardless of whether cells were isolated from peripheral blood, bone marrow, or spleen and regardless of the target cell type.

### 2.2 NK Cells From Old Adults Depict Reduced Conjugation With Senescent Human Dermal Fibroblasts and Leukemic Cells

**FIGURE 2:**
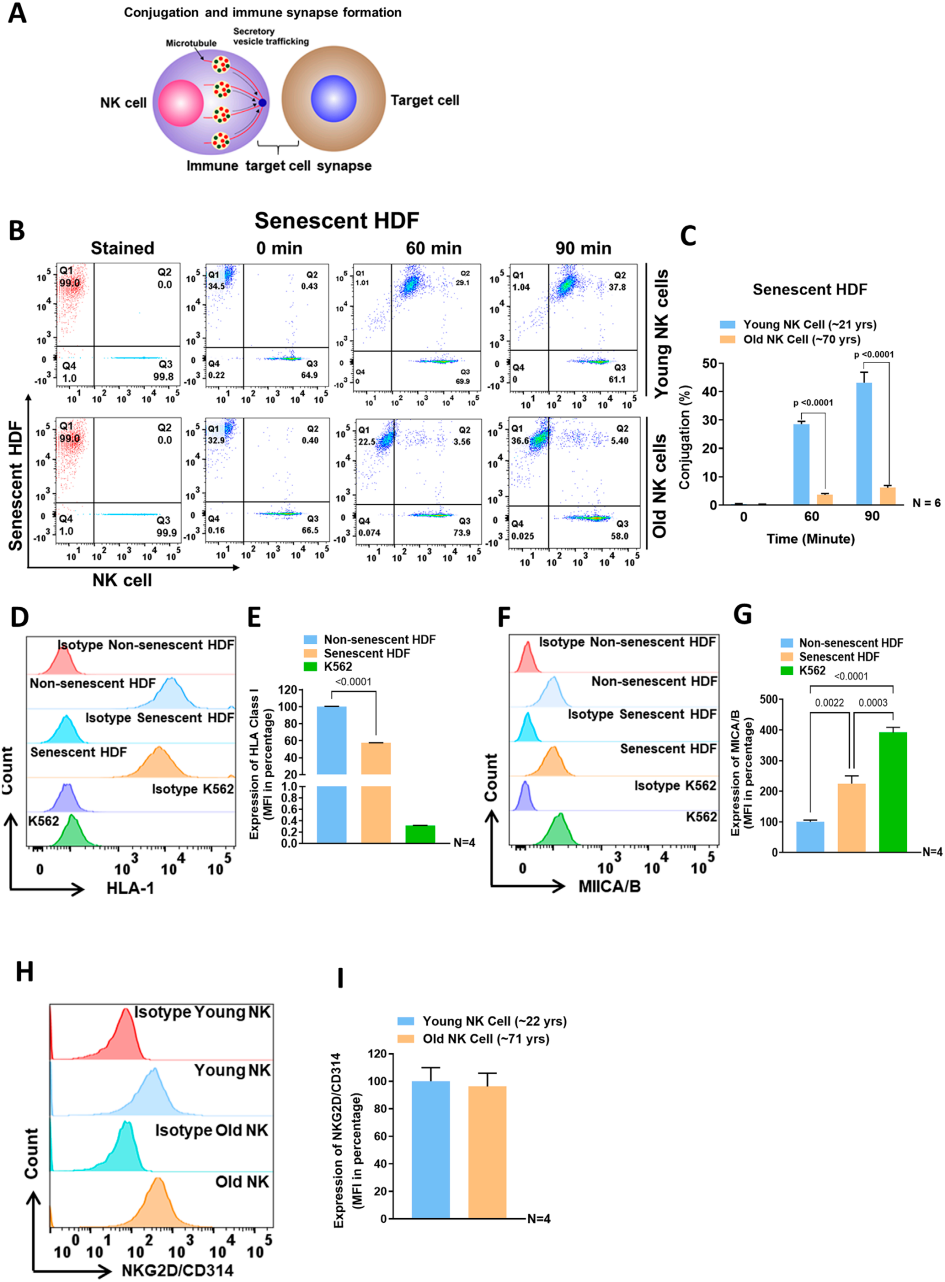
Natural killer cells from old adults depict reduced conjugation with target cells. (A) Scheme illustrating the trafficking of secretory granules within NK cells towards the synapse between NK cells and the corresponding target cells. Synapse formation with the target cell is referred to as conjugation. (B) Flow cytometry analyses of conjugation of NK cells from young (average age 21 years) and old (average age 70 years) donors with senescent HDF following co‐cultures for 0, 60, and 90 min at an effector to target (E:T) cell ratio of 1:1. Senescent HDF were stained with CellTrace violet (450/50 filter), while NK cells from either young or old adults were stained with CellTrace CFSE (530/30 filter). The depicted data set is representative of 6 independent experiments. The dot plot was shown in bi‐exponential scale. (C) Graph shows quantification of the conjugation between either young or old NK cells with senescent HDF at the indicated time points after initiation of co‐cultures (0, 60, and 90 min). Data were presented as mean (conjugation of cells in percentage of total cells) ± SEM, *N* = 6. Two‐way ANOVA, followed by Bonferroni multiple comparison test was used to find the significance between the groups. (D) Flow cytometry analysis of HLA‐1 expression on non‐senescent (young) HDF, senescent HDF and the K562 leukemia cell line. The K562 cells served as conceptual control and IgG1 served as isotype control for each of the groups. (E) Quantification of HLA‐1 expression (MFI) in non‐senescent (young) HDF, senescent HDF and K562. Data were presented as percentage of mean (mean fluorescence intensity, MFI) ± SEM. *N* = 4. Two‐tailed *t*‐test was used to find the significance between non‐senescent and senescent HDF. (F) Flow cytometry analysis of MICA/B expression on non‐senescent (young) HDF, senescent HDF and K562. IgG1 served as isotype control for each of the groups. (G) Quantification of MICA/B expression (MFI) in young and senescent HDF and K562. Data were presented as percentage of mean (MFI) ± SEM, *N* = 4. One‐way ANOVA, followed by Bonferroni multiple comparison test was used to find the significance. (H) Flow cytometry analysis of NKG2D/CD314 expression in NK cells from young and old donors. IgG1 served as isotype control for each of the groups. (I) Quantification of NKG2D/CD314 expression (MFI) on NK cells from either young or old adults. Data in the graphs were represented as percentage of mean (MFI) ± SEM, *N* = 4. Two‐tailed *t*‐test was used to find the significance between NK cells from young and old donors.

Inspired by the impaired interaction of aged NK cells with their target cells (Videos S1 and S2), we sought to further investigate the process of the formation of the synapse that represents the first step in the complex killing process in more detail. NK cells from old adults formed less frequently stable interactions with senescent fibroblasts with a maximum frequency of conjugation of 5.4% vs. 37% for cells from young adults at all time points studied (0, 60, 90 min after initiation of co‐cultures) (Figure 2A–C). Similarly, a significant reduction in the extent of conjugation with leukemic target cells (OSU‐CLL, HG3 and JVM2) was observed for old vs. young NK cells (Figure S4A–F). Reduced MHC‐1 expression marks target cells for removal by NK cells (Elliott et al. 2010). We therefore explored the expression of human histocompatibility leukocyte antigen (HLA) class I molecule (Rajagopalan and Long 1999) and the stress ligand MICA and MICB on target cells (senescent HDF and leukemic cell line K562) and their corresponding activating surface receptors NKG2D/CD314 on young and old NK cells by flow cytometry. Senescent HDF displayed markedly lower HLA‐1 surface expression compared to non‐senescent HDF, with the leukemic cell line K562 showing an even more reduced level. In fact, we found a strong correlation of reduced HLA‐1 expression on senescent HDF and K562 cells (Figure 2D,E) with reduced NK cell mediated killing by NK cells from old adults (Figure 1C,D and Figure S2A,B). We also observed a markedly elevated expression of the stress ligand MHC class 1 chain‐related protein A and B (MICA/B) (Ghadially et al. 2017) on senescent HDF compared to non‐senescent HDF, with even greater levels of expression on K562 cells (Figure 2F,G).

We next investigate whether there was any difference in the corresponding surface receptors (Killer cell immunoglobulin receptor and NKG2D/CD314) on NK cells isolated from young and old individuals. NKG2D/CD314 is one of NK cell activating receptors that recognizes MICA/B ligand on their corresponding target cell (Wang et al. 2024). When compared between NK cells from young and old donors, no significant difference was observed in the expression of NKG2D/CD314 (Figure 2H,I). Even with reduced HLA class I—which should promote killing by reduced interaction of inhibitory receptors recognizing HLA class—and even with no change in NKG2D but increased MICA/B level, the NK cells from old adults show less killing. This means that the classical HLA class I recognizing NK cell receptors as well as the MICA/B‐NKG2D axes do not play an obvious role in this phenomenon of reduced killing.

### 2.3 Reduced Degranulation of Perforin and Granzyme B in NK Cells From Old Adults

**FIGURE 3:**
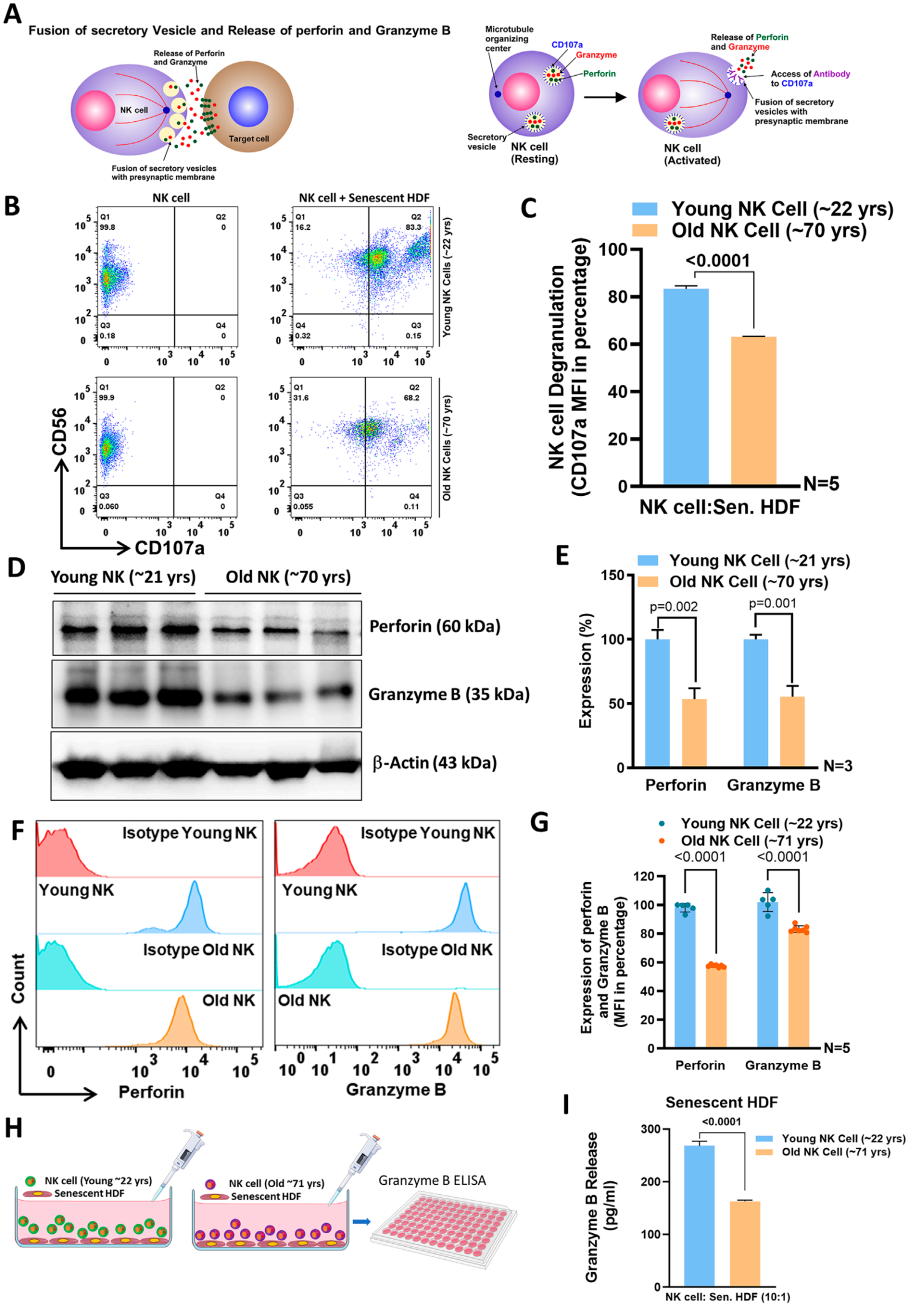
Natural killer cells from old adults reveal impaired degranulation with reduced release of cytolytic perforin and granzyme B. (A) Graphical illustration depicting an important step in the cytotoxicity cascade: Fusion of the vesicles and release of the content of the lytic vesicles (perforin and granzyme B) from NK cells into the immune synaptic cleft (left panel). The fusion of the lytic vesicles with the presynaptic membranes of the NK cells can be followed experimentally by the exposure of CD107a (right graphical panel). (B) Flow cytometry analysis of NK cells from young or old adults depicting exposure of CD107a indicative of the degranulation following co‐culture of NK cells with senescent HDF at an effector to target (E:T) cell ratio of 10:1. Spontaneous degranulation of NK cells without exposure to senescent HDF was used as negative control for degranulation, left dot plots. (C) Quantification of the mean fluorescence intensity (MFI) of the degranulation marker CD107a on NK cells from either young or old adults when co‐cultured with senescent HDF. Data were presented as percentage of mean (mean fluorescence intensity) ± SEM, *N* = 5. Two‐tailed *t*‐test was used to find the significance between the groups. (D) Western blot analysis of perforin and granzyme B content in NK cells isolated from 3 different young and 3 old healthy donors. Actin served as loading control. (E) Graph shows the densitometric quantification of western blots for perforin and granzyme B in young and old NK cells normalized to actin. Data were represented as percentage of mean densitometric value ± SEM, where the expression in NK cells from young donors considered as 100% expression and relative expression in NK cells from old donors were calculated relative to this reference (100%). *N* = 3. Two‐tailed *t*‐test was used to find the significance between NK cells from young and old donors. (F) Flow cytometry analysis of perforin and granzyme B expression in NK cells from young and old human adults. IgG1 served as isotype control for each of the group. (G) Quantification of perforin and granzyme B expression (MFI) in NK cells co‐cultured with either young or senescent HDF. Data were presented as percentage of mean (mean fluorescence intensity) ± SEM, *N* = 5. Two‐way ANOVA, followed by Bonferroni multiple comparison test was used to find the significance. (H) Illustration of the experimental design showing ELISA based measurement of granzyme B release from NK cells in the cell culture supernatant of co‐culture with senescent HDF. (I) Release of granzyme B (pg/mL) from NK cells from either NK cells from old adults or young adults with senescent HDF at an effector to target (E:T) cell ratio of 10:1. Data were represented as mean ± SEM, *N* = 5. Two‐tailed *t*‐test was used to find the significance between the groups.

To further elucidate the underlying defect in the cytotoxic potential of NK cells from old adults, we analyzed whether there are differences between young and aged NK cells in the specific steps of conferring cytotoxicity. After conjugation and immune synapse formation, NK‐cell mediated cytotoxicity continues with the fusion of cytotoxic granule containing vesicles, loaded with perforin and granzyme B, with the presynaptic membrane. This results in the release of these effector molecules into the synaptic cleft between NK and target cells. Perforin perforates the membrane of the target cell with subsequent entrance of granzyme B into the target cell which initiates cell death (Lettau et al. 2007; Pipkin and Lieberman 2007; Chowdhury and Lieberman 2008; Topham and Hewitt 2009). This process can be quantified via the determination of the extent of externalization of lysosome‐associated membrane protein 1 (LAMP‐1) or CD107a, which is normally located on the luminal side of lysosomes (Alter et al. 2004; Dons'koi et al. 2011). There was a significant reduction in degranulation (accessibility of CD107a) of aged NK cells compared to young NK cells (Figure 3A–C). Reduced degranulation of aged NK cells was also observed during co‐cultivation with leukemia cell lines (OSU‐CLL, HG3 or JVM2) (Figure S5A,B). Using Western blot (Figure 3D,E) and flow cytometric analyses (Figure 3F,G), we detected indeed significantly lower levels of perforin and granzyme B in NK cells from older adults compared with those from younger adults. NK cells from older adults also exhibited significantly diminished perforin and granzyme B release in co‐cultures with senescent target cells (Figure S5C, Figure 3H,I). In aggregate, NK cells from old adults suffer from an impaired conjugation and degranulation with insufficient content of perforin and granzyme B and also release of cytotoxic granzyme B.

### 2.4 Globally Altered Transcriptome and Pathways in NK Cells From Old Adults

**FIGURE 4:**
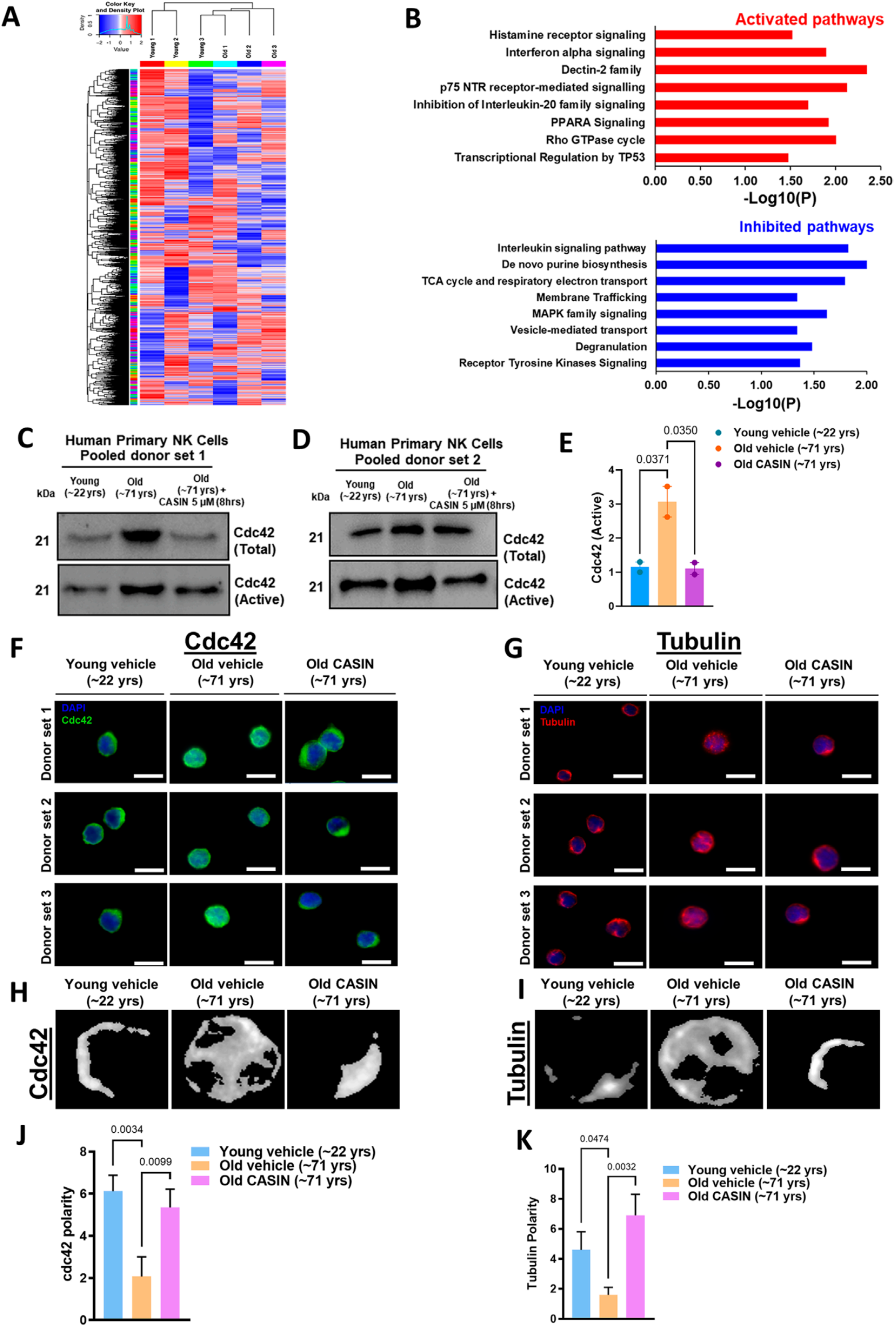
Natural killer cells from old adults show specific changes in the transcriptome and regulation of distinct signaling pathways. (A) Heatmap representing the global gene expression pattern in NK cells isolated from young and old healthy human donors. The color key represents the expression intensity from blue color (low expression) to red color (high expression), while white color represents no expression (0 value). (B) Pathway analyses of transcriptome data of NK cells isolated from young and old donors, based on upregulated and downregulated gene sets. The activated (upper graph) and inhibited (lower graph) pathways in NK cells isolated from old donors in comparison to young donors are presented. (C, D) Pull down western blot analysis of total and active Cdc42 of lysates from NK cells of donor set 1 (C) and donor set 2 (D), each donor set consisting of NK cells from 6 pooled young donors, 6 pooled old donors and 6 pooled CASIN (5 μM) treated NK cells from old donors. (E) Graph showing densitometric analysis of active Cdc42 level in the vehicle treated NK cells from young, old donors and CASIN treated NK cells from old donors. The densitometric value of NK cells from young donor set 1 was set to one fold (reference). The relative densitometric values from other groups relative to reference was then computed and plotted. One‐way ANOVA, followed by Bonferroni multiple comparison test was used to find the significance. (F, G) Representative immunofluorescence staining and distribution of Cdc42 (green) and tubulin (red) in NK cells from young and old donors treated with vehicle, and NK cells from old donors treated with CASIN. Immunostaining was performed on NK cells of 3 different donors per experimental group. (H, I) Representative immunofluorescence micrograph for the quantification of the spatial distribution of Cdc42 (G) and tubulin (H) of young vehicle treated NK cells (first panel), old vehicle treated NK cells (middle panel) and old CASIN treated NK cells (last panel). Fifteen single cells were used for each analysis. (J, K) Quantification of Cdc42 (H) and tubulin (I) polarity score of data shown in F and G, as mentioned in Section 4. Data were represented as mean ± SEM, *N* = 15 individual cells for each group. One‐way ANOVA, followed by Bonferroni multiple comparison test was used to find the significance.

To identify differences in gene expression, we generated whole genome transcriptomic data of NK cells from 3 young and 3 old adults. Heat map analysis showed a large number of genes differentially expressed between young and aged NK cells (Figure 4A). Detailed information on the key genes associated with significantly over‐ and under‐represented pathways, including their known functions, was provided in Tables S3 and S4. Pathway enrichment analyses of differentially expressed gene sets in young and old NK cells revealed that the most up‐regulated pathways included histamine receptor signaling, interferon‐alpha signaling, p53 signaling, PPAR‐alpha signaling, and p75 NTR signaling. Notably, Rho GTPase signaling was also up‐regulated in NK cells from older adults (Figure 4B).

Suppressed pathways in NK cells from old adults included interleukin signaling, MAPK signaling, receptor tyrosine kinase signaling, purine biosynthesis, membrane trafficking with vesicle‐mediated transport and degranulation. Changes in membrane trafficking, vesicular transport and degranulation processes did match with the reduced cytotoxic activity of aged NK cells due to reduced conjugation, vesicle degranulation and the release of granzyme B (Figures 1 and 2). A decline in degranulation of NK cells from old adults is further consistent with a downregulation of gene sets (e.g., SNAP25, MS4A3, SH3RF1, ANK3, ATP11A, NDC80) responsible for the degranulation process in NK cells from old adults (Figure 4B). Furthermore, inactivation of several metabolic pathways, including TCA, the electron transport chain, the de novo purine pathway and respiration, was observed (Figure 4B). In addition, reduced interleukin signaling and MAPK signaling, both involved in NK cell growth and cytotoxicity, were observed. The potential interrelationship between genes of the histamine pathway, the interleukin pathway and Rho GTPase signaling are addressed in the discussion.

### 2.5 CASIN Rebalances Unrestrained Cdc42 Activity in NK Cells From Old Adults

We next sought to identify which pathway(s) might be central for influencing conjugation, synapse formation, degranulation, and potentially also affect mitochondrial function. Due to the previously published role of elevated activity (GTP‐bound form) of the small RhoGTPase *c*ell *d*ivision *c*ontrol protein 42 (Cdc42) in aging and our identification of upregulation of RhoGTPase signaling in aged NK cells, we focused first on Cdc42 signaling. Cdc42 plays an important role in the regulation of microtubular organization as well as the polarity of cells. Elevated activity of Cdc42 causes aging of hematopoietic stem cells (Sinha and Yang 2008; Florian and Geiger 2010; Florian et al. 2012). A focused polar distribution of vesicles containing the cytotoxic cargo in NK cells essentially depends on microtubular organization, and mitochondrial distribution and function also may be regulated by microtubules (Lopez‐Domenech et al. 2018; Cho et al. 2021). We therefore explored whether there is a role of Cdc42 in impaired cytotoxicity in NK cells from old adults. To this end, we determined the activity of Cdc42 in NK cells from young and old adults. We observed higher expression and an elevated level of the active (GTP‐bound) form of Cdc42 in aged compared to young NK cells (Figure 4C–E). Interestingly, treatment of aged NK cells with a specific pharmacological inhibitor of Cdc42 activity (CASIN) (Florian et al. 2012) significantly reduced the unrestrained activation and total Cdc42 expression in NK cells from old donors to the level reported for NK cells from young donors (Figure 4C–E; Figure S6A,B). We then explored whether aged NK cells show disrupted microtubular organization and polarity, and—if so—whether attenuation of Cdc42 activity via CASIN might at least in part rebalance this to a youthful level. NK cells with polarized accumulation of tubulin and Cdc42 are anticipated to be essential for proper conjugation and synapse formation with target cells to initiate the cytotoxicity cascade. Immunofluorescence staining of Cdc42 (green) and tubulin (red) showed polarized distribution of both Cdc42 and tubulin in the NK cells isolated from young individuals (Figure 4F,G, outer left panels). In stark contrast, NK cells from old individuals showed a non‐polarized distribution of Cdc42 and tubulin (Figure 4F,G, middle panels). Of note, CASIN treatment of NK cells from old donors reverted the non‐polarized, diffuse cellular distribution of Cdc42 and tubulin to a more polarized pattern (Figure 4F,G, outer right panels). The polarity scores for Cdc42 and tubulin in NK cells from young donors, and old donors in the presence and absence of CASIN quantitatively confirmed these results (Figure 4H–K).

### 2.6 CASIN Rescues Impaired Conjugation, Vesicle Fusion, and Mitochondrial Function in NK Cells From Old Adults

**FIGURE 5:**
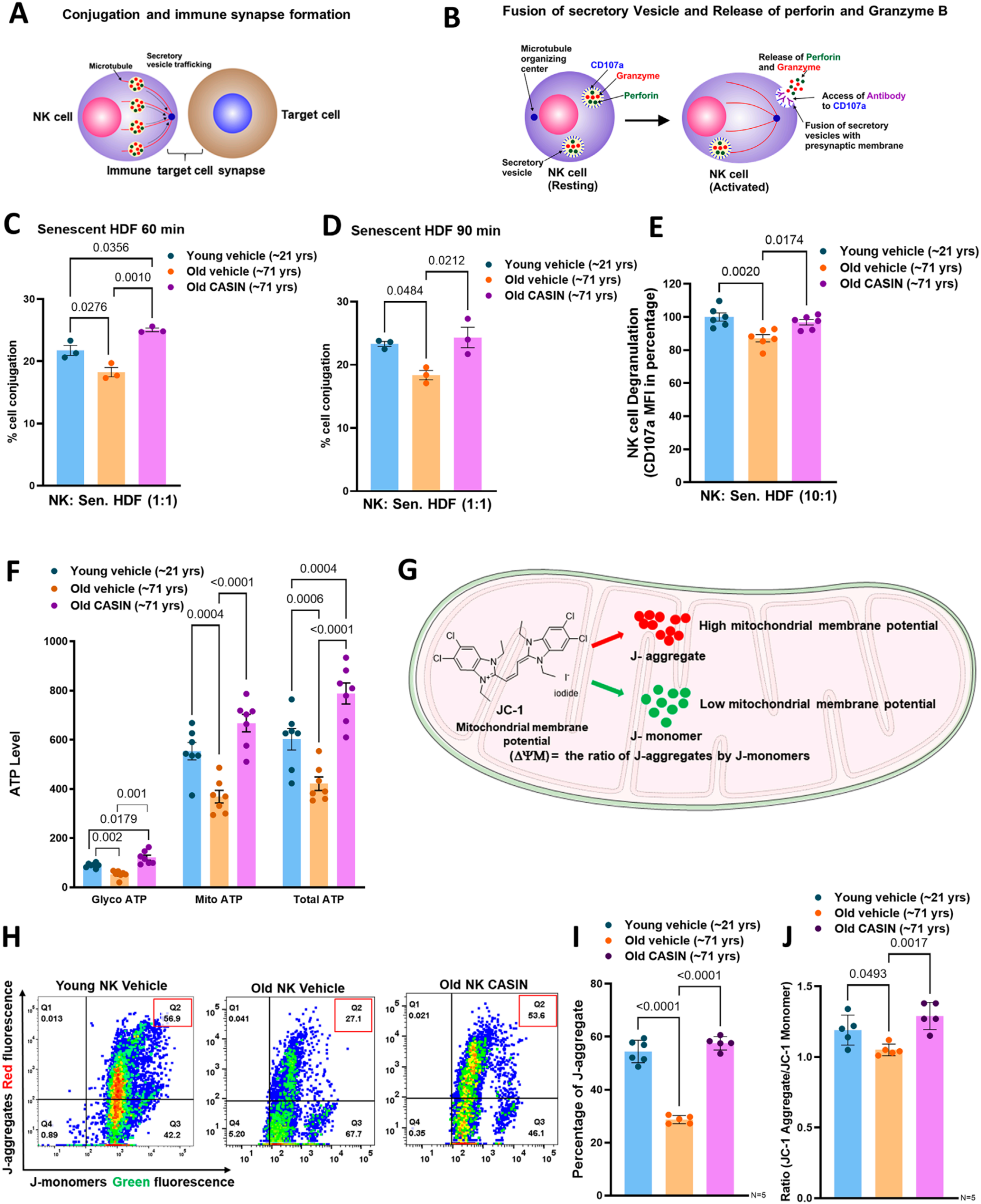
CASIN restores impairment of conjugation, degranulation and mitochondrial ATP generation in old Natural killer cells. (A) Synapse formation with conjugation of NK cells with the target senescent fibroblasts and the tubulin network pulling the perforin and granzyme B containing vesicles in the direction of the synapse. (B) Fusion of the NK cell derived secretory granules with the presynaptic membrane of NK cells and concomitant exposure of CD107a at the cell membrane and the release of perforin and granzyme B into the synaptic cleft towards the target cell. (C & D) Percentage of NK cell conjugation with senescent HDF when co‐cultured for (C) 60 and (D) 90 min at an effector to target (E:T) cell ratio of 1:1. Data were represented as mean (percentage of cell conjugation) ± SEM, *N* = 3. (E) Degranulation of NK cells when co‐cultured with senescent HDF for 7 h at an effector to target (E:T) cell ratio of 10:1. Data were represented as mean (mean fluorescence intensity) ± SEM, *N* = 6. One‐way ANOVA, followed by Bonferroni multiple comparison test was used to find the significance among the groups in C, D and E. (F) Seahorse flux analysis showed quantification of ATP generated by NK cells treated with vehicle from young donors and from NK cells treated with either vehicle or CASIN from old donors. The ATP generation either by glycolysis or by oxidative phosphorylation and total ATP was assessed. Data were represented as mean (ATP level) ± SEM, *N* = 7. Two‐way ANOVA, followed by Bonferroni multiple comparison test was used to find the significance among the groups. (G) Mitochondrial structure showing the chemical structure of the mitochondrial fluorescent probe JC‐1 that can form J‐aggregates (red) and J‐monomers (green) indicating high and low mitochondrial membrane potential, respectively. (H) Flow cytometry analysis of J‐aggregates (red) and J‐monomers (green) of young NK cells treated with vehicle, old NK cells treated with either vehicle or CASIN. (I) The graph depicts the percentage of J‐aggregates (Q2 population of figure H) of young NK treated with vehicle, and old NK cells treated with either vehicle or CASIN. Data were represented as mean (percentage of cell with J‐aggregate) ± SEM, *N* = 6. (J) Quantification of the ratio of J‐aggregates to J‐monomers from young and old NK treated with vehicle and old NK cells treated with CASIN. Data were represented as mean (ratio) ± SEM, *N* = 5. One‐way ANOVA, followed by Bonferroni multiple comparison test was used to find the significance among the groups in I and J.

These findings prompted us to investigate whether CASIN treatment of NK cells from older individuals might also enhance early events in the cytotoxicity cascades thereby improving the impaired killing capacity of NK cells from older donors. Next, we compared conjugation and vesicle fusion (degranulation) of CASIN treated NK cells from old adults to those of vehicle treated NK cells from old and young adults (Figure 5A–E). We found that CASIN treatment significantly rescued the disrupted conjugation (Figure 5C,D) and the impaired degranulation (Figure 5E) in NK cells from old adults when co‐cultured with senescent HDF. The rescue was comparable in terms of conjugation and vesicle fusion to that observed in co‐cultures of senescent HDF with NK cells from young donors. Moderating excessive Cdc42 activation to lower levels restores a younger function of aged NK cells.

Our transcriptome data imply that mitochondrial function, in particular energy generation through oxidative phosphorylation and TCA cycle, might be suppressed in NK cells from old adults (Figure 4A,B). Microtubular organization, vesicle transport and degranulation are energy consuming processes (Mace and Orange 2014), and there is emerging evidence that mitochondrial functions involved in energy production may depend on the balanced microtubular organization (Chen and Chan 2009; Melkov and Abdu 2018). We therefore explored whether ATP production and mitochondrial polarization are compromised in NK cells from old adults and whether CASIN can revert this possible mitochondrial dysfunction. Seahorse flux analysis showed reduced total ATP levels (composed of glycolytic ATP and mitochondria generated ATP) in NK cells from old adults. CASIN treatment significantly enhanced total ATP levels in old NK cells (Figure 5F). ATP synthesis by mitochondria is regulated by mitochondrial membrane potential. Active mitochondria show high membrane potential (hyperpolarization), due to pumping out of H^+^ by the proton pumps of the electron transport chain. To assess the polarization of the mitochondrial membrane, we employed JC‐1 fluorescence measurement. At high potential JC‐1 aggregates within the mitochondrial matrix (red fluorescence) and at low potential JC‐1 stays as monomers (green fluorescence) (Figure 5G). NK cells from old adults revealed a significantly lower percentage of J aggregates (Figure 5H,I) and a reduced ratio of JC‐1 aggregates/JC‐1 monomers (Figure 5J), compared to NK cells from young adults indicating a lower membrane potential. Of note, CASIN treatment of NK cells from old adults led to a significant increase of the membrane potential of aged NK cells (Figure 5I,J).

### 2.7 CASIN Treatment Improved Killing Ability of NK Cells From Old Humans and Mice

**FIGURE 6:**
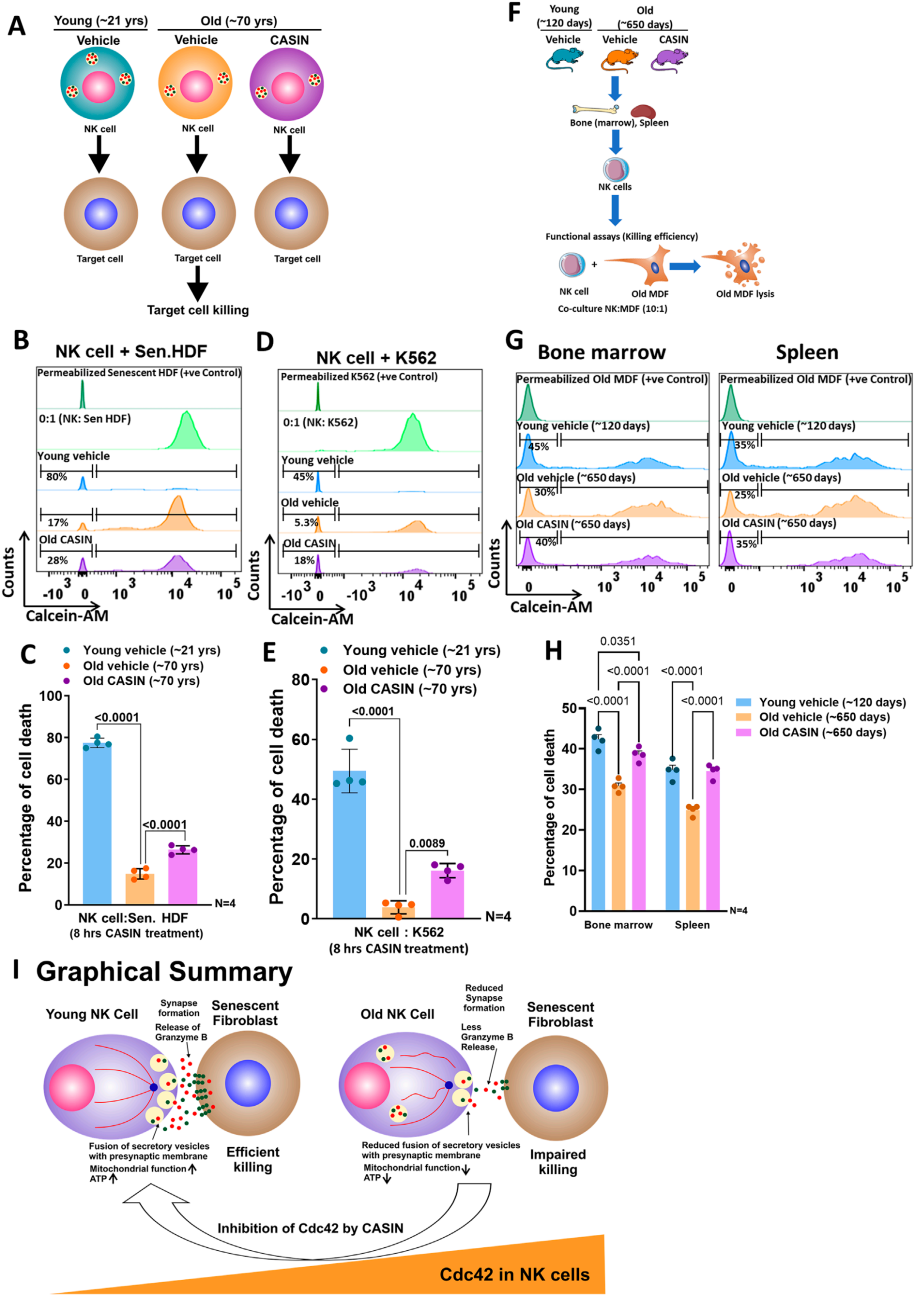
CASIN treatment improves the cytotoxic ability of Natural killer cells from old humans and mice. (A) Graphical illustration of experimental plan, where young NK cells treated with vehicle and old NK cells treated with either vehicle or CASIN for 8 h and thereafter subjected to co‐culture with target senescent HDF exerting their differential killing ability. (B) Representative histograms depicting the killing ability of different experimental groups as measured by flow cytometry. Peak at the left side of histogram, showing the dead senescent HDF population with percentage of dead cells. (C) Quantification of the percentage of target senescent HDF death executed by young NK cells treated with vehicle, and old NK cells treated with either vehicle or CASIN. Data were represented as mean (Percentage of senescent fibroblast death) ± SEM. *N* = 4. (D) Representative histograms show the distribution of K562 killing by young NK cells treated with vehicle, and old NK cells treated with either vehicle or CASIN. Peak at the left side of histogram, showing the dead K562 population with percentage of dead cells. (E) Graph shows the percentage of target cell (K562) death mediated either by young NK cells treated with vehicle or by old NK cells treated with either vehicle or CASIN. Data were represented as mean (Percentage of K562 lysis) ± SEM. *N* = 4. One‐way ANOVA, followed by Bonferroni multiple comparison test was used to find the significance among the groups in C and E. (F) Illustration of the experimental design for treatment of young mice (average age 120 days) treated with vehicle and old mice (average age 650 days) treated with either vehicle or CASIN. Following treatment, NK cells were isolated from spleen and bone marrow and subjected to co‐cultures with murine dermal fibroblasts (MDF) derived from old mice (average age 650 days). (G) Flow cytometry with representative histograms depicting old/senescent MDF killing by NK cells isolated from bone marrow (left panel) and spleen (right panel) of vehicle and CASIN treated old mice. Peak at the left side of histogram, showing the dead old MDF population with percentage of dead cells. (H) Quantification of the percentage of old/senescent MDF killing by NK cells isolated from bone marrow and spleen of young and old mice treated with vehicle and old mice treated with CASIN. Data were represented as mean (percentage of old/senescent MDF lysis) ± SEM, *N* = 4, where each group contains pool of NK cells isolated from 4 different mice of same treatment group. Two‐way ANOVA, followed by Bonferroni multiple comparison test was used to find the significance among the groups. (I) Graphical summary. Unrestrained Cdc42 activity causes failure of old NK cells to kill senescent fibroblasts. Unrestrained Cdc42 activity disrupts the microtubular network and impaired mitochondrial ATP resulting in reduced conjugation, and impaired degranulation of lytic vesicles into the synaptic cleft with reduced cytotoxicity. CASIN can attenuate all these steps and in part attenuate the killing of senescent fibroblasts (senescent HDF).

To finally explore whether the Cdc42 activity inhibitor CASIN also restored impaired cytotoxicity of aged NK cells, both in vitro and in vivo approaches were used (Figure 6). Flow cytometry revealed a significant enhancement in the cytotoxicity on senescent HDF mediated by old NK cells treated in vitro with CASIN (Figure 6A–C). CASIN‐treated NK cells from older adults further showed a marked increase in migration velocity and more efficient killing of senescent fibroblasts in co‐cultures (Video S3) compared with non‐treated NK cells from older adults (Video S2). A similar result was observed, with NK cells from older adults treated with CASIN showing significantly enhanced killing of the leukemic cell line K562 compared to vehicle‐treated NK cells from the same age group (Figure 6D,E). In addition, in vivo treatment of old mice with CASIN (Figure 6F) resulted in rejuvenated NK cell mediated cytotoxicity towards old MDF (Figure 6G,H) and YAC‐1 (Figure S6C–E), when compared with NK cells isolated from vehicle treated mice. Both bone marrow and spleen derived NK cells showed improved killing potential following treatment of CASIN in vivo (Figure 6F–H and Figure S6C–E). These data indicate that the age‐related loss of human NK cell mediated cytotoxicity against senescent fibroblasts and other target cells shares underlying mechanisms with NK cells from aged mice, and that in both species this impairment can be restored to a more youthful level by attenuation of Cdc42 activity via CASIN.

To address whether in vivo Cdc42 inhibition by CASIN affects the number of resident NK cells in the skin during aging, we performed immunofluorescence staining of skin sections from vehicle‐ and CASIN‐treated young and old mice to detect NK cells (NK1.1, green). No significant differences were observed in the number of resident NK cells between vehicle‐ and CASIN‐treated groups (Figure S7A,B), indicating that CASIN primarily influences NK cell function in aged mice rather than their abundance.

## 3 Discussion

Our major finding is that overactivation of the small Rho GTPase Cdc42 severely impairs the cytotoxicity cascade of NK cells from old adults which, in consequence, fail to efficiently kill senescent fibroblasts and other target cells (Figure 6I, graphical summary). We found that age‐related loss of NK cell cytotoxicity is likely due to a whole cascade of events that includes impaired immune synapse formation (conjugation), reduced degranulation, reduced release of cytotoxic granules, and diminished ATP generation. Interestingly, a specific inhibitor of the activity of Cdc42 (CASIN) disrupted conjugation, reduced perforin and granzyme B release, and mitochondrial dysfunction and enhanced ATP generation, eventually improving the killing ability (Figure 6I). CASIN binds to Cdc42 and attenuates Cdc42 activity by reducing the exchange of GDP with GTP on the protein.

The observed impairment in NK‐cell cytotoxicity from old adults has substantial clinical implications, as it likely promotes the accumulation of senescent fibroblasts and malignant or virus‐infected target cells, thereby driving aging and aging‐related diseases (Maity et al. 2021; Wlaschek et al. 2021; Ogrodnik et al. 2024; Bohm et al. 2025). It also increases the susceptibility to infections, and facilitating malignant tumor progression, including hematological malignancies (Ovadya et al. 2018; Fane and Weeraratna 2020).

A likely reason for the reduced adhesion and synapse formation observed under hyperactivated Cdc42 is impaired microtubule organization and actin polymerization. Enhanced Cdc42 activity thus may hinder the reliable formation of a planar NK‐cell membrane surface, a prerequisite for effective conjugation with target cells. Cdc42 has been reported to promote the formation of membrane filopodia (Nobes and Hall 1995; Krugmann et al. 2001). Overactivation of Cdc42 may thus enhance increased frequency and quantity of micro spike formation (small membrane protrusions) which likely disturb NK cell adhesion/conjugation to target cells. Enhanced Cdc42 activity with the disruption of the microtubular organization may also lead to an impaired fusion of granules and vesicles with the presynaptic membrane with reduced perforin release. This notion is supported by the attenuation of the inefficient degranulation of NK cell by CASIN which rebalances Cdc42 activity and the polarity of microtubules. Previous data supports our finding that translocation of the lytic granules to the synaptic cleft depend on an organized microtubular network (Huse 2012). Our work thus fills the gap between reduced cytotoxic ability of NK cells from old adults and the upstream mechanism that unrestrained Cdc42 activation is critically contributing to impaired cytotoxicity in NK cells from old adults.

Inspired by unbiased bulk transcriptome analysis depicting suppressed pathways in mitochondrial metabolism, we here provide direct experimental evidence that ATP levels are reduced in NK cells from old adults, and can be restored by CASIN, thereby readjusting Cdc42 activity. Currently the relationship between enhanced Cdc42 activity and mitochondrial dysfunction with reduced ATP generation is poorly understood. Previous reports suggest that microtubules are highly important for mitochondrial trafficking, intracellular distribution and function (Chen and Chan 2009; Melkov and Abdu 2018). In the case of Cdc42 hyperactivity, we show that the polarized distribution of tubulin, a main component of microtubular network, is disrupted and this may directly impact on mitochondrial function. Even though, the evidence provided is in part correlative, a reduction in ATP levels as shown in NK cells from old adults will impact on energy demanding processes such as synapse formation, vesicle transport, and the release of the cytotoxic cargo. This is further supported by data from our bulk transcriptome analysis and pathway enrichment analysis unveiling a profound reduction in vesicle transport, degranulation and the ATP generating process of mitochondrial oxidative phosphorylation. CASIN, specifically inhibiting Cdc42 overactivation in NK cell from old adults almost completely reverses all steps of the cytotoxicity cascade and mitochondrial dysfunction and increased ATP levels to that of NK cells from young adults. These data highlight the important role of Cdc42 in the cytotoxicity cascade and the killing ability of NK cells from old adults.

Currently, the up‐stream mechanisms resulting in hyperactivation of Cdc42 in aging, and aging‐related diseases are not well understood. Several possibilities may contribute and need to be further addressed in future. Paracrine or autocrine SASP factors from old NK cells or senescent cells in the neighborhood of NK cells may induce Cdc42 activity. SASP factors like Interleukin‐1 (IL‐1) and Tumor Necrosis Factor‐α (TNF‐α) have been reported to induce Cdc42 activity (Puls et al. 1999). Alternatively, changes in the composition of the extracellular matrix with reduced osteopontin was associated with an enhanced activity of Cdc42 in HSC (Guidi et al. 2017). Also, posttranslational modifications, such as phosphorylation and ubiquitination can regulate Cdc42 activity (Rossman et al. 2005). Previously, the histaminylation of Cdc42 resulting in the inhibition of GTP hydrolysis eventually leading to an unrestrained activation of Cdc42 was reported in mast cells (Vowinckel et al. 2012). Interestingly, the histamine pathway is the most up‐regulated pathway in aged NK cells, and it remains to be elucidated in future whether this enforces enhanced Cdc42 activity leading to impaired NK cell cytotoxicity and the accumulation of senescent cells in tissues and organs. Likewise, we found mRNA specifically coding for TEC, a receptor‐independent tyrosine kinase, to be reduced in aged NK cells, and this was earlier reported to occur in conditions of unrestrained Cdc42 activation (Takesono et al. 2004). TEC is important for the regulation of interleukins also of those which enhance NK cell cytotoxicity. The complex interrelationship between TEC, Cdc42 activity and interleukin release needs to be further explored in aged versus young NK cells.

Our study does not allow to rule out the contribution of factors other than Cdc42 to at least in part to improve the NK cell cytotoxicity following CASIN treatment. While our data highlight the role of Cdc42 in regulating NK cell cytotoxicity, we cannot exclude the contribution of additional mechanisms. For instance, improved cellular bioenergetics may enhance the metabolic fitness of NK cells, thereby supporting sustained cytotoxic responses. Likewise, more efficient immune synapse formation could facilitate stronger and more stable interactions between NK cells and their targets, promoting an improved delivery of cytotoxic granules. Other age‐related changes in signaling pathways, membrane dynamics, or cytoskeletal organization independent of Cdc42 may also play a role. Thus, the observed improvement in NK cell function following CASIN treatment is likely multifactorial, with Cdc42 acting in concert with complementary processes that collectively augment cytotoxic activity.

We here focused on the mechanism underlying reduced killing ability of NK cells of senescent fibroblasts driving aging. In addition, we provide evidence that NK cells from old adults cannot efficiently kill a variety of malignant leukemic cells from patients suffering from chronic myeloid leukemia or chronic lymphatic leukemia, suggesting that the mechanisms unveiled herein with reduced NK cell killing ability applies not only for the removal of senescent fibroblasts, but is more general, and may also be exploited for therapeutic intervention in aging and aging‐related malignancies. In perspective, this will allow to develop new avenues for therapeutic interventions to prevent and possibly treat aging, and aging‐related diseases including among others hematological malignancies, diseases widely occurring at old age.

## 4 Materials and Methods

### 4.1 Resources Table

**Table:** 

Reagent or Resource	Source	Identifier
*Antibodies and dyes*		
Antihuman CD56‐FITC	Biolegend	Cat. #362546
Antihuman CD107a‐APC	Biolegend	Cat. #328620
Antihuman perforin	Cell Signaling	Cat #62550S
Antihuman Perforin	Biolegend	Cat #308132
Antihuman Granzyme B	Cell Signaling	Cat #44153S
Antihuman/mouse Granzyme B	Biolegend	Cat #372226
Antihuman p21	Cell Signaling	Cat. #2947
Antihuman p16INK4a	R&D Systems	Cat. #AF5779
Antihuman CD107a‐APC	Biolegend	Cat. #328620
Antihuman cdc42 detection kit	Cell Signaling	Cat. #8819
Antimouse NK1.1	Invitrogen	Cat. #14‐5941‐82
Antimouse CD3	Abcam	Cat. #ab5690
Antihuman/mouse Cdc42	ThermoFisher Scientific	Cat. #PA1‐092
Antihuman HLA Class I	Biotechne	Cat. #FAB7098P
Antihuman MICA/B	Biolegend	Cat. #320908
Antihuman NKG2D/CD314	Biolegend	Cat. #320806
Antihuman Tubulin	Sigma‐Aldrich	Cat. #T9026‐100UL
Antimouse p16INK4a	Abcam	Cat. #Ab211542
Calcein Green AM dye	Invitrogen	Cat. #C34852
Calcein Red AM dye	Invitrogen	Cat. #C34851
CellTrace CFSE	Invitrogen	Cat. #C34570
CellTrace violet	Invitrogen	Cat. #C34571
Fc Receptor Blocking Solution	Biolegend	Cat. #422302
JC‐1 fluorescent dye	Cell Signaling	Cat. #92891
*Primary cells and cell lines*		
Chronic lymphocytic leukemia cells	Comprehensive Cancer Centre, Ulm University	
Human dermal fibroblast	iXCells Biotechnologies	Cat. #10HU‐013
Human dermal fibroblast	ATCC	Cat. #ATCC‐PCS‐201‐010
Human dermal fibroblast	Lifeline	Cat. #FC‐0001
Natural killer cells	DRK‐Blutspendedienst Ulm, Baden‐Württemberg, Germany	Buffy Coat
Human skin biopsies	Department of Dermatology and Allergic Diseases, University Hospital, Ulm University	
HG‐3	Comprehensive Cancer Centre, Ulm University	
JVM‐2	Comprehensive Cancer Centre, Ulm University	
K562 cell line	ATCC	Cat. #CCL‐243
OSU‐CLL	Comprehensive Cancer Centre, Ulm University	
YAC‐1	ATCC	Cat. #TIB‐160
*Software and Instrument*		
BD LSRFortesser Cell Analyzer	BD Bioscience	Cat. #649225B7
CellDetail software	Matlab	Schuster, Amoah et al., 2024
Fiji		https://fiji.sc/
Flowjo software	BD Bioscience	
Fusion FX7 Imager	Vilber Laurmat, Germany	
Prism software	GraphPad	
Seahorse software	Agilent Technologies	
Zeiss Axioster plus inverted microscope	Zeiss	
EasyEight EasySep magnet	StemCell	Cat. #18103
Collagenase A	Roche	Cat #10103586001
CASIN	Cayman Chemical	Cat. #17694
Citric acid	Sigma‐Aldrich	Cat. #77–92‐9
Doxorubicin	Sigma‐Aldrich	Cat. #D1515‐10MG
EasySep human NK cell isolating kit	Stem Cell	Cat. #17955
EasySep mouse NK cell isolating kit	Stem Cell	Cat. #19855
FCCP	Sigma‐Aldrich	Cat. #C2920
Human granzyme B DouSet Elisa Kit	R&D Systems	Cat #DY2906‐05
LumiGlo/Peroxide	Cell Signaling	Cat. #7003
Magnesium chloride (MW95.21)	Sigma‐Aldrich	Cat. #7786‐30‐3
NEBNext Ultra II Directional RNA Library Prep Kit	NEB	Cat. #E7760L
Oligomycin	Agilent Technologies	
Potassium ferricyanide (MW329.2)	Sigma‐Aldrich	Cat. #13746–66‐2
Potassium ferrocyanide (MW422.4)	Sigma‐Aldrich	Cat. #14459–95‐1
RNA Isolating/RNeasy plus mini Kit	Qiagen	Cat. #74004
Rotenone/antimycin	Agilent Technologies	
Sodium Chloride (MW58.44)	Sigma‐Aldrich	Cat. #7647‐14‐5
Sodium phosphate (MW163.94)	Sigma‐Aldrich	Cat. #7601‐54‐9
ß‐Actin SC	Santa Cruz	Cat. #130301
X‐Gal	Bioline	Cat. #BIO‐37035
*Medium and reagent*		
Alpha‐MEM	ThermoFisher Scientific	Cat. #22561021
Dimethul Sulfoxide	Sigma‐Aldrich	Cat. #D8418
DMEM	Gibco, UK	Cat. #41965039
Fetal Bovine Serum (FBS)	Sigma‐Aldrich	Cat. #S0615
Heat inactivated horse serum	ThermoFisher Scientific	Cat. #26050088
IL‐2/proleukin S	Novartis	Cat. #171520000
L‐glutamine	PAN‐Biotech	Cat. #P0480100
Pancoll human separating solution	Pan Biotech	Cat. #P04‐66500
Penicillin/streptomycin	PAN‐Biotech	Cat. #P0607100
RPMI 1640	ThermoFisher Scientific	Cat. #21875034

### 4.2 Isolation and Culture of Human Primary NK Cells

Buffy coat from healthy human young (18–25 years) and old (69–75 years) donors were provided by the Institute for Clinical Transfusion Medicine and Immunogenetics, Ulm University (DRK‐Blutspendedienst Ulm, Baden‐Württemberg, Germany). Peripheral blood mononuclear cells (PBMCs) were isolated by Ficoll density gradient centrifugation method using Pancoll human separating solution (Pan Biotech, Cat. #P04‐66500). Thereafter, NK cells were further isolated from PBMCs using the NK cell isolating kit (Stem cell, Cat. #17955) as described in the manufacturer's instructions, and purified NK cells were then resuspended in complete NK cell growth media, which comprises of 75% alpha‐MEM (Thermo, Cat. #22561021), 12.5% heat‐inactivated horse serum (Thermo, Cat. #26050088) and Fatal Bovine serum (Sigma Aldrich, Cat. #S0615), 2 mM L‐glutamine (PAN‐Biotech, Cat. #P0480100), 10,000 U/mL penicillin/10 mg/mL streptomycin (PAN‐Biotech, Cat. #P0607100), and 5 ng/mL IL‐2/proleukin S (Novartis, Cat. #171520000) and cultured at 37°C and 5% CO_2_. All NK cells isolated in this study were first cultured for 24 h in complete NK cell growth media before further use.

### 4.3 Human Dermal Fibroblasts Isolation and Culture

Three different neonatal foreskins derived human dermal fibroblasts (HDF) were used in this study. Donor 1 was purchased from iXCells Biotechnologies (Cat. #10HU‐013); other fibroblasts donor cells were purchased from ATCC (Cat. #ATCC‐PCS‐201‐010) and Lifeline (Cat. #FC‐0001). HDF was cultured in complete DMEM medium (10% FBS, 1× Pen/Strep, 1× L‐glutamate) in a humidified 5% CO_2_, 95% atmospheric air and 37°C. Upon confluency of 80%–90%, the cells were passaged into 1:5 and further cultured.

### 4.4 Senescence Induction in Human Dermal Fibroblasts

Replicative senescence was induced by repeated passaging of HDF (1 × 10^6^ in 175 cm^2^ flask with 20 mL complete DMEM media) until desired cumulative population doubling (CPD) was reached. The CPD was calculated as described earlier (Silva et al. 2016). HDF (1 × 10^6^ in 175 cm^2^ flask with 20 mL complete DMEM media) were treated with doxorubicin (Sigma Aldrich, Cat. #D1515‐10MG) at a dose of 500 nM for 72 h to induce senescence. Also, HDF (1 × 10^6^ in 175 cm^2^ flask with 20 mL complete DMEM media) were exposed to gamma irradiation at a dose of 10Gy to induce senescence.

### 4.5 Human Skin Biopsies

Human skin biopsies (Table S1) from healthy donors were provided by the Department of Dermatology and Allergic Diseases, University Hospital, Ulm University. For fibroblast isolation, skin samples were first placed in ice cold DMEM and cut into tiny pieces. After brief washing with DMEM, 5 mL DMEM with collagenase A (2 mg/mL) was added and incubated at 37°C and 5% CO_2_ with 2–3 times pipetting to achieve proper skin digestion. Cells were then centrifuged at 1500 rpm for 10 min; the pellets were resuspended in complete DMEM medium and cultured only for low passages.

### 4.6 Human Primary Chronic Lymphocytic Leukemia Cells

Chronic lymphocytic leukemia (CLL) cells were isolated, characterized (Table S2), and provided by the Comprehensive Cancer Centre, Ulm University.

### 4.7 Human Chronic Myelogenous Leukemia and CLL Cell Lines

K562 cell, a chronic myelogenous leukemia cell line, was purchased from ATCC (ATCC, Cat. #CCL‐243). OSU‐CLL, HG‐3 and JVM‐2: OSU‐CLL and HG‐3 are chronic lymphocytic leukemia cell lines, while JVM‐2 is a mantle cell lymphoma cell line that were characterized and cultured as described earlier (Rosen et al. 2012; Hertlein et al. 2013). These cell lines were cultured in RPMI medium 1640 (Thermo, Cat. #21875034) with 10% FBS, 1% L‐Glutamine and 1% Pen/Strep as supplements, at a temperature of 37°C and 5% CO_2_.

### 4.8 Murine Skin Biopsies

Young (average age 100 days) and old (average 700 days) wild type (WT) C57BL/6J mice were purchased from Charles River Laboratory and kept under approved conditions in the mouse facility at Ulm University in accordance with the animal welfare protective law. All murine experiments are confirmed by the ethical committee at Ulm University and are approved by the Regierungspräsident Tübingen, Germany. Murine dermal fibroblasts (MDF) were isolated and cultured as previously described (Maity et al. 2021).

### 4.9 NK Cell Mediated Target Cell Cytotoxicity Assay

Human dermal fibroblasts, old mouse dermal fibroblasts, primary hematological leukemic cells, hematological leukemic cell lines (K562, OSU‐CLL, HG3, JVM2) and YAC‐1 (ATCC, Cat. #TIB‐160) were subjected to vital staining with Calcein AM dye (Invitrogen, Cat. #C34852) for 30 min at 37°C and 5% CO_2_. For this 1 × 10^6^ respective cells were resuspended in 1 mL PBS containing 1 μM calcein AM dye. After staining, the cells were washed two times with 1× PBS. NK cells isolated from peripheral blood of young and old human donors as well as bone marrow or spleen of young and old mice were cultured for 24 h prior to co‐culture with the indicated target cells. Co‐cultures were performed in 24‐ or 48‐well plates for 7 h at 37°C in a humidified 5% CO_2_ atmosphere using complete NK cell culture medium, as described above. For each coculture 5 × 10^5^ calcein stained respective target cells and NK cells as required (i.e., 5 × 10^5^ for 1:1 ratio, 5 × 10^6^ for 10:1 ratio) were added. Following incubation, cells were collected and flow cytometry was conducted to assess NK cell mediated cytotoxicity of target cells. Cell death was quantified by measuring Calcein fluorescence intensity at 530 nm (excitation at 488 nm) using an LSR Fortessa cytometer. Minimum 10,000 events were recorded for flow cytometric analyses. Each assay was done in replicates (3–5 per group). Analyses of flow cytometric data were performed by FACSDIVA and Flowjo. The peak of the histograms were presented as modal and axes in bi‐exponential scale.

### 4.10 NK Cell and Target Cell Conjugation Assay

NK cells (1 × 10^6^/mL in PBS) from young and old human adults were stained with CellTrace CFSE (Invitrogen, Cat. #C34570) and different target cells (1 × 10^6^/mL in PBS) such as, senescent HDF, K562, OSU‐CLL, HG3 and JVM2 were stained with CellTrace violet (Invitrogen, Cat. #C34571) for 20 min at 37°C and 5% CO_2_ environment. Following staining, complete culture media (1 mL) was added to the staining reaction, followed by gentle mixing and cell counting. Cells were then centrifuged at 1500 rpm for 5 min, and cell pellets were resuspended in complete NK cell culture media. NK cells (1 × 10^6^) from young and old human adults were cocultured with senescent HDF (1 × 10^6^) in FACS tubes at an effector to target (E: T) cell ratio of (1:1) in 100 μL:100 μL culture media for 0, 20 and 30 min at 37°C and 5% CO_2_. NK cells (1 × 10^6^) were also co‐cultured separately with OSU‐CLL, HG3 or JVM2 (1 × 10^6^) in FACS tubes at an effector to target (E: T) cell ratio of (1:1) in 100 μL:100 μL culture media for 0, 10, 20 and 30 min at 37°C and 5% CO_2_. Thereafter, the tube was vortexed for 3 s and subjected to flow cytometry (LSR Fortessa, BD) analysis using excitation at 405 nm and emission at 450/50 nm filter set, while for CFSE excitation at 488 nm and emission at 530/30 nm filter set. A minimum of 10,000 events were recorded for flow cytometric analyses. Analyses and visualization of flow cytometric data were performed by FACSDIVA and Flowjo. The axes in the dot plots were presented in bi‐exponential scale.

### 4.11 NK Cell Degranulation Assay

Young and old NK cells (1 × 10^6^) were co‐cultured separately with different target cells (1 × 10^5^), such as senescent human dermal fibroblasts and hematological cancer cells (OSU‐CLL, HG3 and JVM2) at an effector to target (E: T) cell ratio of (10:1) for 7 h at 37°C and 5% CO_2_. Young and old NK cells (1 × 10^6^) without any target cell were used as negative control. After incubation with target cells, the cells in the co‐culture were washed with PBS and stained for 10 min with Fc Receptor Blocking Solution (Biolegend Cat. #422302) at 4°C. The cells were then stained with CD56‐FITC (Biolegend Cat. #362546) and CD107a‐APC (Biolegend Cat. #328620) for 30 min at 4°C. After washing two times with FACS buffer, cells were resuspended in 300 μL FACS buffer and subjected to flow cytometry (LSR Fortessa, BD) analysis using excitation at 488 nm and emission at 530/30 nm filter set and excitation at 640 nm and emission at 670/14 nm filter set. Minimum 10,000 events were recorded for flow cytometric analyses. Analyses of flow cytometric data were performed by Flowjo (version 10). The axes in the dot plots were presented in bi‐exponential scale.

### 4.12 Flow Cytometry of Surface Receptors and Intracellular Proteins

Expression of HLA‐1, MICA/B on HDF (non‐senescent and senescent), K562 and NKG2D/CD314 on NK cells from young and old donors was studied by flow cytometry as mentioned previously above. In brief, 1 × 10^6^ NK cells from young or old donors in 100 μL FACS buffer were incubated with 5 μL of fluorescent labeled antibody for 30 min at 4°C. After washing two times with FACS buffer, cells were resuspended in 300 μL FACS buffer and subjected to flow cytometry (LSR Fortessa, BD) as described earlier. For intracellular staining, such as perforin, granzyme b, NK cells (1 × 10^6^) were first fixed and permeabilized with fixation/permeabilization buffer using Foxp3/Transcription Factor Staining Buffer Set kit (Thermo). Afterwards, fixed and permeabilized NK cells were incubated with 5 μL of fluorescent labeled antibody for 30 min at room temperature, as described in the manufacturer protocols. Following final washing, NK cells were resuspended in permeabilization/wash buffer and flow cytometry was performed as described earlier. Analyses of flow cytometric data were performed by FACSDIVA and Flowjo (version 10). The peak of the histograms was presented as modal and axes in bi‐exponential scale.

### 4.13 Perforin Release Assay by Flow Cytometry

Release of perforin from NK cells when co‐cultured with senescent HDF was measured by indirect way using flow cytometry. The intracellular content of perforin was measured in NK cells at different time points of co‐culture. NK cells (1 × 10^6^) from young and old donors were separately co‐cultured with senescent HDF (1 × 10^5^) in NK cell culture media (400 μL) and at different time point of co‐culture (0, 2, 4 and 6 h). NK cells were collected from co‐culture, centrifuged and fixed to stain perforin, followed by flow cytometric measurement as described earlier. The content of perforin at 0 h of co‐culture in the NK cells from young donors was set as 100% perforin content. The perforin content in other samples in comparison with this reference point served as perforin content of that particular sample at that particular time point. Any decrease in perforin content of a particular NK cell sample from 0 h was considered as perforin release. The MFI of each sample was subtracted from the respective isotype control.

### 4.14 Granzyme B Release

Release of granzyme B from NK cells was measured by sandwich ELISA (R&D, Cat. #DY2906). Young and old NK cells (1 × 10^6^) were co‐cultured with senescent HDF (1 × 10^5^) in 24 well plate with 300 μL of NK cell culture media [E: T cell ratio of 10:1] for 7 h at 37°C and 5% CO_2_. Following incubation, cell culture supernatants were collected from different NK cell and senescent HDF co‐cultures. After centrifugation, cell culture media (supernatants) were used for assessment of granzyme B concentrations according to the manufacturer's instructions. In brief, 100 μL of clear cell culture medium was added to each well and incubated at room temperature for 2 h, after aspiration of cell culture media and three times washing with wash buffer 100 μL of detection antibody was added and incubated further for 2 h at room temperature. Thereafter, the detection antibody was discarded followed by three times washing with washing buffer. Subsequently, 100 μL of substrate solution was added to each well, followed by 20 min incubation at room temperature. 50 μL of sulfuric acid stop solution was next added to each well and the optical density was measured at 450 nm. The amount of granzyme B in each sample was then calculated from the standard curve.

### 4.15 Western Blot Analysis

Western blots were performed on NK cells and human dermal fibroblasts, respectively. In brief, cells were lysed with ice‐cold RIPA lysis buffer (25 mM Tris–HCl (pH 7.6), 150 mM NaCl, 1% NP‐40, 1% sodium deoxycholate, 0.1% SDS) with protease and phosphatase inhibitor cocktail. The estimated protein concentration for each sample lysate is determined using the Bradford assay (Bio‐Rad). Equal amounts of protein are loaded into the 4%–20% SDS‐PAGE gel. The proteins were then transferred onto a nitrocellulose membrane overnight at 4°C. The membrane was blocked for 1 h in 5% BSA at room temperature. Primary antibodies for NK cell, perforin (Cell Signaling, Cat #62550S) and Granzyme B (Cell Signaling, Cat #44153S), as well as human dermal fibroblasts, p21 (Cell Signaling, Cat. #2947) and p16INK4a (R&D Systems, Cat. #AF5779) were diluted in BSA 1:1000 and added onto the membrane and incubated overnight at 4°C on a shaker. After 3 washing steps in 1× PBS, the indicated secondary antibodies (conjugated to horseradish peroxidase) were separately added at a concentration to the incubation buffer and western membranes were incubated therein at room temperature for 1 h. Finally, the different membranes were incubated for 2–3 min in the dark with prepared chemiluminescent substrate solution (20× LumiGlo reagent and 20× Peroxide, Cell Signaling, Cat. #7003) and images were taken using the Fusion FX7 imager (Vilber). The densitometric values of respective bands were performed using Fusion Bio1D software (Vilber). Each band intensity (perforin or granzyme B) was normalized to the corresponding densitometric value of β‐actin, which served as the loading control. The normalized values for perforin and granzyme B in young NK cells were set as 100%. Relative to these values, the normalized expression levels in old NK cells were converted to a percentage scale. For Cdc42 (active) the densitometric value in young NK cells was set as 1. Relative to these values, the densitometric value of Cdc42 (active) in old NK cells was converted.

### 4.16 RNA Isolation, Library Preparation and Transcriptome Sequencing

Total RNA was extracted from young and old primary NK cells (1 × 10^6^ each) using RNeasy kit according to the manufacturer's instructions (Qiagen). For transcriptome analysis, 1 μg of total RNA from young and old NK cells was first rRNA depleted using either Ribominus Eukaryotic system v2 kit (ThermoFisher Scientific). rRNA depleted RNA was subjected to preparation of Illumina compatible RNA‐seq libraries using NEBNext Ultra II Directional RNA Library Prep Kit (NEB, Cat #E7760L). The libraries were then quality controlled through Qiaxcel advanced system (Qiagen) measured by Qubit fluorimeter (ThermoFisher Scientific). Validated libraries were next sequenced on Illumina Novaseq 6000 system (MLL, Munich) using S1 flow cells and 2 × 100 cycles. The demultiplexed fastq files were next used for data analyses. The sequencing reads were first adapter trimmed and quality filtered. The quality filtered fastq reads were next aligned to human reference genome (GRCh38) using Hista2 with dta‐cufflinks option. The aligned reads were then used for transcriptome assembly using cufflinks and finally differential expression of genes was measured by cuffdiff. Pathway analyses were performed by Ingenuity Pathway Analysis (Qiagen) and Reactome pathway as described earlier (Maity et al. 2021). Visualization and representation of the transcriptome data were performed by R scripts in R‐Studio environment.

### 4.17 Pull Down Assay of Cdc42 in NK Cells

The active cdc42 detection kit (Cell Signaling Technology Cat. #8819) was used in the cdc42 pull down assay and was done accordingly as stated in the manufacturer's protocol. Briefly, stimulated NK cells (10 × 10^6^) or treated with either CASIN or DMSO were subjected to the pull down of active cdc42 using cell signaling cdc42 detection kit. 500 μg of pooled cell lysate for each group was used for the assay.

### 4.18 Immunofluorescent Staining of Paraffin Section

5 μm thick paraffin skin sections were first warmed at 60°C for 30 min. Thereafter deparaffinized in xylene for 2 × 5 min, rehydrated in a decreasing alcohol gradient and water, followed by antigen unmasking in a hot 10 mM sodium citrate buffer pH 6 for 10–15 min. Sections were washed with 1× PBS three times for 5 min each and then blocking in 5% BSA and 10% goat serum for 1 h at room temperature. At 4°C, an overnight incubation of primary antibody of NK cells, anti NK1.1 (Cat. #14‐5941‐82, 1:200, Invitrogen) and T‐cells, anti CD3 (Cat. #ab5690, 1:200, Abcam) was done. After 3 × 5 min each washing with 1× PBS, the sections were incubated for 1 h with the respective fluorochrome conjugated secondary antibody (1:1000, Alexa 555, red and Alexa 488, green, both Invitrogen) at room temperature in the dark. Before pictures were taken using the Zeiss Axioster plus Inverted Microscope, the sections were incubated for 3 min in DAPI.

### 4.19 Immunofluorescent Staining for Cdc42 and Tubulin Polarity in NK Cells

NK cells were first fixed in 4% PFA for 10 min and transferred on the glass slides using the thermo scientific Cytospin 4 centrifuge (program 3, rpm 250 and acceleration low) for 5 min. Cells were then treated with 0.1% Triton X‐100 for 10 min at room temperature followed by 3× 5 min each washing with 1× PBS and 1 h blocking in 5% BSA and 10% goat serum. Primary antibodies, Cdc42 (Cat. #PA1‐092, 1:200, Thermo) or Tubulin (Cat. #T9026‐100UL, 1:200, sigma) was incubated overnight at 4°C. Sections were incubated for 1 h with the respective fluorochrome conjugated secondary antibody (1:1000, Alexa 555, red and Alexa 488, green both Invitrogen) at room temperature in the dark, after 3× 5 min each washing with 1× PBS. Finally, for staining the cell nucleus, the sections were incubated for 3 min in DAPI and pictures were taking using the Zeiss Axioster plus Inverted Microscope. Quantitative determination of the spatial distribution of components (cdc42 and tubulin) in single cells was performed with CellDetail software (Schuster et al. 2024).

### 4.20 Seahorse Extracellular Flux Assay

Freshly isolated NK cells (1 × 10^6^) were first cultured in NK cell culture medium overnight, and then treated with either vehicle (DMSO) or 5 μM CASIN for 8 h and washed 2× with 1× PBS. The glycolysis and OXPHOS of NK cells were measured according to the Agilent XF seahorse manufacturer's instructions. In short, 250,000 cells/well were seeded in a coated Poly‐L‐Lysine 96 well plate in Agilent XF RPMI (10 mM glucose, 2 mM glutamine and 1 mM pyruvate) recommended medium to determine the oxygen consumption rate (OCR) and extracellular acidification rate (ECAR). The metabolic modulators, oligomycin (1 μM), FCCP (2 μM) and rotenone/antimycin (0.5 μM) were injected accordingly during the assay. Data analysis was done using the Agilent XF Seahorse Analytics software.

### 4.21 In Vivo CASIN Treatment in Mouse

Young and old healthy wild type C57BL/6J mice were injected with either 25 mg/kg of Cdc42—inhibitor CASIN (Cayman chemical, Cat. #17694) or vehicle (cyclodextrin) for 4 consecutive days. Three days after the last injection, the mice were sacrificed, skin and NK cells from spleen and bone marrow were isolated using the EasySep Mouse NK cell isolating kit (Stem cell, Cat. #19855) as described in the manufacturer's instructions. The skin and NK cells were used for immunofluorescence staining and cytotoxicity assay, respectively.

### 4.22 Assessment of Mitochondrial Membrane Potential

After overnight culturing, NK cells (1 × 10^6^) were treated with either CASIN or vehicle for 8 h followed by 2× washing with PBS. 1 million cells for each experimental group were resuspended in 1 mL NK cell medium without FBS and horse serum. For decoupling control, cells were incubated in the presence of 2 μM FCCP (Sigma‐Aldrich, Cat. #C2920) for 30 min. Afterwards, cells were incubated at 37^o^C and 5% CO_2_ for 30 min in 2 μM of JC‐1 fluorescent dye (Cell Signaling, Cat. #92891). Finally, flow cytometry was done to detect J‐aggregate at red fluorescence emission at approximately 590 nm and J‐monomers at green fluorescence emission at a maximum of 529 nm. A minimum of 10,000 events were recorded for flow cytometric analyses. Analyses of flow cytometric data were performed by FACSDIVA and Flowjo (version 10).

### 4.23 Time‐Laps of NK Cell Mediated Killing of Senescent Human Dermal Fibroblasts

Senescent fibroblasts (5 × 10^4^) were cultured for 12 h before the assay on a Cellview cell culture disc (Greiner, Cat. #627860). NK cells (after overnight culture) and senescent fibroblasts were then stained with Calcein red (Invitrogen, Cat. #C34851) and Calcein green AM (Invitrogen, Cat. #C34852) respectively for 30 min. Thereafter, NK cells were added onto the senescent fibroblasts at an effector to target (E:T) cell ratio of 10:1 and co‐cultured for 5 h at 37°C and 5% CO_2_. During this time, a time‐lapse video was made using the Leica TCS SP8 Inverse confocal microscope.

### 4.24 Statistical Analysis

GraphPad Prism (GraphPad Software) was used for the calculation of all the data and then presented as mean ± standard error of mean (SEM). Also, the unpaired two‐tailed Student's *t*‐test for comparing the difference between two samples was used to calculate the statistical significance (*p*‐value). For comparing more than two samples, the one‐way ANOVA or two‐way ANOVA, followed by Bonferroni correction, was employed. The exact test used and the corresponding P‐value of comparison are presented in the respective figure legends and figures.

## Author Contributions

Conceptualization: K.S.‐K. and P.M. Methodology: A.K.K., P.M., K.S. Investigation: A.K.K., P.M., K.S., L.K., Y.W., R.P.D., V.S. Writing – original draft: A.K.K., P.M. Writing – review and editing: K.S.‐K., P.M., A.K.K., K.S., P.H., R.P.D., K.S.‐K., D.F., H.S., S.S., L.W., H.G. Project administration: M.W.

## Funding

The work is funded by the German Research Foundation under the projects DFG, SCHA411/18‐1 “The role of pro‐oxidant connective tissue in skin aging” and CRC1506; Project‐ID 450627322 “Aging at Interfaces” (German Research Foundation). KSK is also supported by CRC1149 (Project‐ID 251293561) “Danger Response, Disturbance Factors and Regenerative Potential after Acute Trauma”, Regenerative Potential After Acute Trauma and by the Graduate Training Group GRK 1789: Project‐ID 194266605 “Cellular and Molecular Mechanisms in Ageing (CEMMA)”.

## Conflicts of Interest

The authors declare no conflicts of interest.

## Supporting information


**Table S1:** Healthy human skin biopsy.
**Table S2:** Human primary chronic lymphocytic leukemia (CLL) cells.
**Table S3:** Activated pathways and gene sets in NK cells from old donors.
**Table S4:** Inactivated pathways and gene sets in NK cells from old donors.

**Figure S1:** Characterization of human and murine senescent dermal fibroblasts and Natural killer cell purity isolated from young and old donors. (A) Representative flow cytometric dot plot of NK cells isolated from young and old individuals stained with anti CD3 for all T cells (in quadrant 1) and anti CD56 for NK cells (in quadrant 3). (B) Graph depicts the percentage of NK cell purity isolated from the blood of young and old adults. Data were represented as mean ± SEM, *N* = 4. (C) Senescence‐Associated β‐galactosidase (SA‐β‐gal) activity, a robust senescence marker, was detected in non‐senescent (young) HDF with a Cumulative Population Doubling (CPD) of 10 and senescent HDF with a CPD > 60. (D) Depicts the quantification of β‐galactosidase positive senescent human dermal fibroblasts compared to non‐senescent (young) HDF per high power field (HPF). A two‐tailed *t*‐test was used to find the significance between the groups. (E) Western blot analysis showing p21 and p16INK4a expression from lysates of young and senescent HDF. Actin served as a loading control. (F) Densitometric quantification of the senescence markers depicted as the ratio of p21 and p16INK4a expression in young and senescent HDF normalized to the actin loading control. Data were represented as mean ± SEM, *N* = 4. A two‐way ANOVA, followed by a Bonferroni multiple comparison test, was used to find the significance among the groups. (G) SA‐β‐gal staining of young (50 days) and old (650 days) murine dermal fibroblasts (MDF) isolated from the back skin of C57Bl/6J mice. (H) Quantification of SA‐β‐gal positive MDF isolated from young and old mice from the results in (G). Data were represented as mean ± SEM, *N* = 4. A two‐tailed *t*‐test was used to find the significance between the groups. (I) Western blot analysis of p16INK4a expression in MDF from young and old mice with actin as a loading control. (J) Quantification of the expression of p16INK4a in MDF. Data were represented as mean ± SEM, *N* = 3. (K) Western blot analysis of p21 expression in MDF with actin as a loading control. (L) Quantification of the expression of p21 in MDF of young and old mice normalized to the actin loading control. Data were represented as mean ± SEM, *N* = 3. A two‐tailed *t*‐test was used to find the significance between the groups in (K) and (L). (M) Senescence‐Associated β‐galactosidase (SA‐β‐gal) detection in non‐senescent, doxorubicin and gamma irradiated HDF from two different donors. (N & O) Quantification of SA‐β‐gal positive cells in non‐senescent, doxorubicin, and gamma irradiated HDF per high power field (HPF). A one‐way ANOVA, followed by a Bonferroni multiple comparison test, was used to find the significance among the groups in (N) and (O). (P) Western blot analysis of p21 and p16INK4a expression from lysates of non‐senescent, doxorubicin and gamma irradiation induced senescent HDF with actin as a loading control. (Q & R) Densitometric quantification of the senescence markers depicted as the ratio of p21 (Left graph) and p16INK4a (Right graph) expression in non‐senescent, doxorubicin and gamma irradiation induced senescent HDF normalized to the actin loading control. Data were represented as mean ± SEM. *N* = 3. A two‐tailed *t*‐test was used to find the significance between the groups in each donor set.
**Figure S2:** Natural killer cell mediated cytotoxicity towards human leukemic cell lines. (A) Flow cytometry analysis of NK cell induced cytotoxicity towards human leukemic cell lines. (B) Graph depicts the percentage of killing of target cells (K562, OSU‐CLL, HG3 and JVM2) by NK cells from young and old human adults at an effector to target (E:T) cell ratio of (10:1). Data are represented as mean ± SEM, *N* = 5. (C) Flow cytometry analysis of NK cell induced cytotoxicity towards primary chronic lymphocytic leukemia (CLL) cells from five different patients diagnosed with different ranges of CLL mutations. (D) Graph indicating the percentage of target cell death against NK cell to chronic lymphocytic leukemia (CLL) cells from five different donors with an effector to target (E:T) cell ratio of 10:1. Donor ID is pseudonymized and indicated as sample1‐51. Data are represented as mean ± SEM, *N* = 5. Two‐way ANOVA, followed by Bonferroni multiple comparison test was used to find the significance among the groups in C and D.
**Figure S3:** Reduced cytotoxicity of the Natural killer cell from old mice towards YAC‐1. (A) Illustration of the experimental design for studying the cytotoxic potential of NK cells from bone marrow and spleen from young and old C57BL6/J mice. NK cells were isolated from bone marrow and spleen and subjected to co‐cultures with a murine lymphoma cell line (YAC‐1). (B) Quantification of the percentage of YAC‐1 killing/death by NK cells isolated from bone marrow and spleen of young and old mice. Data were represented as mean (Percentage of YAC‐1 cell death) ± SEM. *N* = 3. Each mouse NK cell sample used in the cytotoxicity assay was pooled from NK cells isolated from three different mice. Two‐way ANOVA, followed by Bonferroni multiple comparison test was used to find the significance among the groups.
**Figure S4:** Impaired conjugation between Natural killer cells from old human adults and leukemic cell lines. Flow cytometric analysis of the percentage of conjugation between NK cells from young (average age 21 years) and old donors (average age 70 years) and distinct leukemic cell lines with the corresponding quantification as follows: (A, B) conjugation with HG3; (C, D) conjugation with JVM2; (E, F) conjugation with OSU‐CLL. The target leukemic cells were stained with CellTrace violet and NK cells from young and old adults were stained with CFSE, both dyes for living cells. A representative data set is shown for each leukemic target cell. Q2 populations represent NK cells conjugated with leukemic cells. Data were represented as mean (percentage of total cells used in co‐culture) ± SEM, and statistical significance was calculated using unpaired two‐tailed *t*‐test. *N* = 4. Two‐way ANOVA, followed by Bonferroni multiple comparison test, was used to find the significance among the groups in B, D, and F.
**Figure S5:** Reduced degranulation by Natural killer cell from old adults towards hematological cancer cell lines. (A) Flow cytometric analysis of CD107a indicative for lytic vesicle fusion with the external membrane of NK cells and, in consequence, for degranulation of NK cells from young and old donors following co‐culture with the leukemic cell lines OSU‐CLL, HG3 or JVM2 at an effector to target (E:T) cell ratio of 10:1 for 7 h. (B) Shows quantification of the mean fluorescence intensity (MFI) of CD107a (MFI in percentage, where young NK cells were considered as 100%) of young and old NK cells when co‐cultured with OSU‐CLL, HG3 or JVM2. Data were represented as mean ± SEM, N = 5. Two‐way ANOVA, followed by Bonferroni multiple comparison test was used to find the significance among the groups. (C) Shows quantification of the MFI of perforin content (MFI in percentage, where young NK cells at 0 h were considered as 100%) in NK cells from young and old donors when co‐cultured with senescent human dermal fibroblasts at different time points of co‐culture. Data were represented as mean ± SEM, N = 5. Two‐way ANOVA, followed by Bonferroni multiple comparison test was used to find the significance among the groups.
**Figure S6:** Increased Cdc42 activity in Natural killer cells from old donors and improvement of cytotoxicity of NK cells from old mice by CASIN. (A, B) Pull down western blot analysis of total and active Cdc42 activity of lysates from NK cells of pooled donor set 1 (A) and pooled donor set 2 (B) with their respective ß‐Actin. Each donor set consisting of NK cells from 6 pooled young donors, 6 pooled old donors and 6 pooled CASIN (5 μM) treated NK cells from old donors. (C) Illustration of the experimental design with treatment of young mice (120 days) treated with vehicle (DMSO) and old mice (650 days) treated with either vehicle (DMSO) or with CASIN at a concentration of 5 μM/kg for 4 consecutive days. Three days after the last treatment, NK cells were isolated from spleen and bone marrow and subjected to co‐cultures with a murine lymphoma cells cell line (YAC‐1). (D) Flow cytometry with representative plots depicting YAC‐1 killing by NK cells isolated from bone marrow (left panel) and spleen (right panel) of young vehicle, vehicle and CASIN treated old mice. (E) Quantification of the percentage of YAC‐1 killing/death against NK cells isolated from bone marrow and spleen of young and old mice treated with vehicle and/or with CASIN at a concentration of 5 μM with an effector to target (E:T) cell ratio of 10:1. Data were represented as mean (Percentage of YAC‐1 death) ± SEM. N = 3. Each mouse NK cell sample used in the cytotoxicity assay was the pool of NK cells isolated from three different mice. Two‐way ANOVA, followed by Bonferroni multiple comparison test was used to find the significance among the groups.
**Figure S7:** No alteration in the numbers of resident Natural killer cells in skin of old mice, following CASIN treatment. (A) Immunofluorescence staining of skin sections with an antibody against NK cells (green) and DAPI (blue). Skin derived from young mice (115 days) treated with vehicle, old mice (650 days) either treated with vehicle or treated with 5 μM CASIN. (B) Quantification of positive NK cells expressed as number per HPF in all experimental groups. Data were represented as mean ± SEM and statistical significance was calculated using an unpaired two‐tailed *t*‐test. *N* = 5, and scale bar = 50 μm. One‐way ANOVA, followed by Bonferroni multiple comparison test was used to find the significance among the groups in C and D.

**Video S1:** acel70398‐sup‐0003‐VideoS1.mp4.

**Video S2:** acel70398‐sup‐0004‐VideoS2.avi.

**Video S3:** acel70398‐sup‐0005‐VideoS3.mp4.

## Data Availability

The raw reads and gene expression matrix files of transcriptome analyses, which were generated and used in this manuscript are available in GEO (GSE313986). Other data that support the findings of this study are available from the corresponding author upon reasonable request.
