# Supplementary material for: Overactivation of Cdc42 GTPase Impairs the Cytotoxic Function of NK Cells From Old Individuals Towards Senescent Fibroblasts
Source: Aging Cell. 2026 Feb 8;25(2):e70398. doi: 10.1111/acel.70398 (PMC12883145; doi:10.1111/acel.70398)
Supplement: Supplementary file 1 — Table S1: Healthy human skin biopsy. Table S2: Human primary chronic lymphocytic leukemia (CLL) cells. Table S3: Activated pathways and gene sets in NK cells from old donors. Table S4: Inactivated pathways and gene sets in NK cells from old donors. [file ACEL-25-e70398-s005.pdf]

Table S1

Sample ID	Position	Gender	Age (yrs)
K93	Under arm	Male	74
K104	Lower abdomen	Male	74
K107	Back	Male	73
K111	Back	Female	70
K101	Lower back	Male	77
K95	Lumba	Male	87

Table S1. Healthy human skin biopsy

Healthy human skin samples indicating donor pseudonymized codes, the specific area on the body where the skin biopsy was taken, gender and age (years) of donor.

Table S2

Sample ID	IGHV status	p53	Chromosomal abberrations
Sample 1	VH1, 1-18, 88.89% mutated 17.05.2011	Unmutated 22.02.2017	Del (13q14.3) 24.05.2013
Sample 2	V4-34 98% mutated	Unmutated	Del (13q14.3)
Sample 3	VH3 3-23 92.16% mutated 28.07.2015	Unknown	Del 13q14.3
Sample 4	Unknown	c.428T>C 95%wt p.(Val143Ala) 01.04.2015	Del (13q14.3) 20.02.2012
Sample 5	Mutated	Unmutated	Normal Karyotype

Table S2. Human primary chronic lymphocytic leukaemia (CLL) cells

This table provides information about donors with sample ID, immunoglobulin heavy chain variable status, p53 and chromosomal aberration status in chronic lymphocytic leukemia (CLL). Some information is not available (unknown).

Table S3

Activated pathways	Most upregulated genes	Functions
Rho GTPase cycle	CDC42	Small Rho GTPase; regulates actin cytoskeleton, cell polarity, migration, and vesicle trafficking.
	TCTEX1D1	Dynein light chain–related protein; implicated in microtubule motor/dynein complex regulation and intracellular transport.
	OPHN1	Rho-GTPase activating protein (GAP); regulates synaptic morphogenesis, actin cytoskeleton and endocytosis.
Histamine receptor signaling	PRKACB	Catalytic subunit of PKA (protein kinase A); mediates cAMP-dependent phosphorylation signaling.
	GNAS	G protein alpha subunit (Gsα); stimulates adenylate cyclase, cAMP production and diverse GPCR signaling.
	EGR1	Zinc-finger transcription factor; immediate-early gene controlling growth, differentiation and stress responses.
Dectin-2 family	CLEC6A	C-type lectin receptor (dendritic cell receptor); pattern recognition in innate immunity and antigen uptake.
	CLEC10A	C-type lectin (endocytic receptor); mediates glycan recognition, antigen uptake and immune modulation.
Transcriptional Regulation by TP53	CDKN1A	p21 (Cip1); cyclin-dependent kinase inhibitor controlling cell cycle arrest and DNA damage response.
	GADD45A	Stress response protein; DNA repair, cell cycle arrest, apoptosis and maintenance of genomic stability.
	TP53	p53 tumor suppressor; transcriptional regulator of DNA damage response, apoptosis and cell cycle arrest.
	NUAK1	AMPK-related kinase; regulates cell adhesion, metabolism, stress response and cell motility.

Table S3. Activated pathways and gene sets in NK cells from old donors

Table S4

Inactivated pathways	Most downregulated genes	Functions
Degranulation	SNAP25	SNARE protein; mediates synaptic vesicle docking/fusion and neurotransmitter exocytosis.
	MS4A3	Membrane-spanning 4A family member; implicated in hematopoietic cell signaling and cell cycle regulation.
	SH3RF1	E3 ubiquitin ligase/adapter (SH3 domain-containing); involved in receptor trafficking and JNK signaling.
	ANK3	Ankyrin-G; scaffolding protein linking membrane proteins to cytoskeleton, important in polarity and membrane domain organization.
	ATP11A	P4-ATPase phospholipid flippase (membrane asymmetry); translocates aminophospholipids across membranes.
	NDC80	Kinetochore complex component; essential for chromosome alignment and microtubule attachment during mitosis.
Membrane Trafficking	CKM	Creatine kinase; catalyzes reversible transfer of phosphate between creatine and ATP (energy buffering).
	KIF28P	Kinesin family member; likely microtubule motor–related.
	MKRN3	E3 ubiquitin ligase (Makorin family); implicated in puberty timing and ubiquitin-dependent regulation.
	SYT8	Synaptotagmin family member; calcium-sensing regulator of vesicle exocytosis/endocrine secretion.
	TRPM4	Ca2+-activated nonselective cation channel; regulates membrane depolarization and Ca2+ signaling indirectly.
	FNBP1L	F-BAR domain protein (formin-binding); involved in endocytosis, actin dynamics and membrane remodeling.
Vesicle-mediated transport	MKRN3	mentioned above
	SYT8	mentioned above
	TRPM4	mentioned above
	FNBP1L	mentioned above
	SFN	14-3-3σ (stratifin); cell cycle regulator and scaffold protein involved in checkpoint control and signaling.
	USP6NL	Rab5 effector with GAP activity (TBC/RABGAP); regulates endocytosis and receptor trafficking.
	KIF27	Kinesin family motor protein; involved in intracellular transport and ciliary/hedgehog pathway regulation.
	ANK3	mentioned above
	RALGAPA2	Ral GTPase activating protein subunit; negative regulator of Ral signaling and vesicle trafficking.
The citric acid (TCA) cycle and respiratory electron transport	MT-ND2	Mitochondrial NADH dehydrogenase 2 (Complex I subunit); electron transport chain and oxidative phosphorylation.
	MT-ND1	Mitochondrial NADH dehydrogenase 1; Complex I subunit in oxidative phosphorylation.
	MT-ND3	Mitochondrial NADH dehydrogenase 3; Complex I subunit in electron transport.
MAPK family signaling cascades	PAK3	p21-activated kinase 3; effector of CDC42/RAC1 controlling cytoskeleton, neuronal development and signaling.
	PANX2	Pannexin family channel protein; ATP release and intercellular signaling, expressed in brain.
	RAC3	Rho family GTPase; regulates actin cytoskeleton, cell migration, and signaling to proliferation/survival.
	GFRA2	GDNF family receptor alpha-2; co-receptor for RET signaling in neuronal survival and differentiation.
	CCND1	Cyclin D1; cell cycle regulator promoting G1/S transition via CDK4/6 activation.

Table S4. Inactivated pathways and gene sets in NK cells from old donors
