# Supplementary material for: Overactivation of Cdc42 GTPase Impairs the Cytotoxic Function of NK Cells From Old Individuals Towards Senescent Fibroblasts
Source: Aging Cell. 2026 Feb 8;25(2):e70398. doi: 10.1111/acel.70398 (PMC12883145; doi:10.1111/acel.70398)
Supplement: Supplementary file 2 — Figure S1: Characterization of human and murine senescent dermal fibroblasts and Natural killer cell purity isolated from young and old donors. (A) Representative flow cytometric dot plot of NK cells isolated from young and old individuals stained with anti CD3 for all T cells (in quadrant 1) and anti CD56 for NK cells (in quadrant 3). (B) Graph depicts the percentage of NK cell purity isolated from the blood of young and old adults. Data were represented as mean ± SEM, N = 4. (C) Senescence‐Associated β‐galactosidase (SA‐β‐gal) activity, a robust senescence marker, was detected in non‐senescent (young) HDF with a Cumulative Population Doubling (CPD) of 10 and senescent HDF with a CPD > 60. (D) Depicts the quantification of β‐galactosidase positive senescent human dermal fibroblasts compared to non‐senescent (young) HDF per high power field (HPF). A two‐tailed t‐test was used to find the significance between the groups. (E) Western blot analysis showing p21 and p16INK4a expression from lysates of young and senescent HDF. Actin served as a loading control. (F) Densitometric quantification of the senescence markers depicted as the ratio of p21 and p16INK4a expression in young and senescent HDF normalized to the actin loading control. Data were represented as mean ± SEM, N = 4. A two‐way ANOVA, followed by a Bonferroni multiple comparison test, was used to find the significance among the groups. (G) SA‐β‐gal staining of young (50 days) and old (650 days) murine dermal fibroblasts (MDF) isolated from the back skin of C57Bl/6J mice. (H) Quantification of SA‐β‐gal positive MDF isolated from young and old mice from the results in (G). Data were represented as mean ± SEM, N = 4. A two‐tailed t‐test was used to find the significance between the groups. (I) Western blot analysis of p16INK4a expression in MDF from young and old mice with actin as a loading control. (J) Quantification of the expression of p16INK4a in MDF. Data were represented as mean ± SEM, N = 3. (K) Western [file ACEL-25-e70398-s003.pdf]

Figure S1

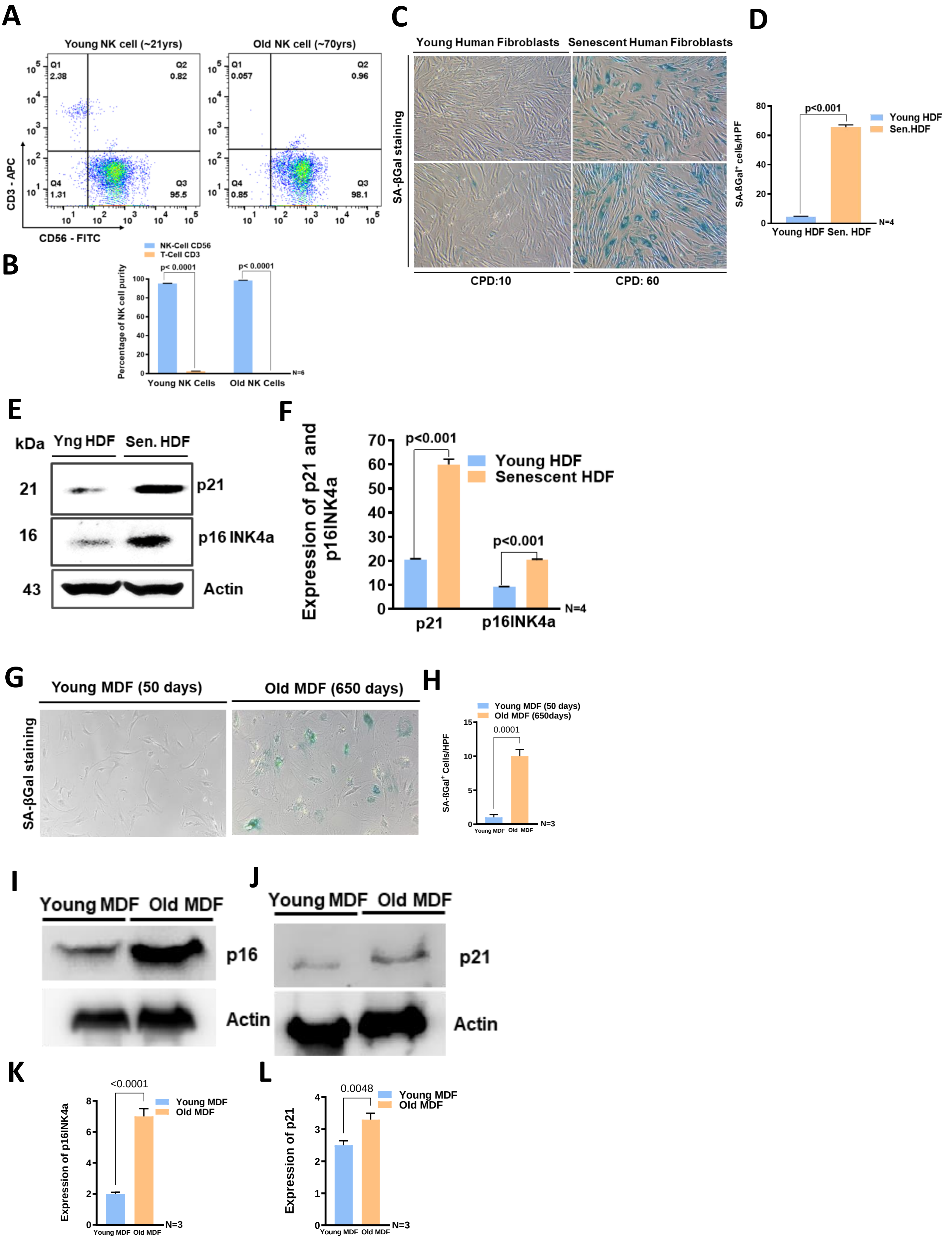

A
Young NK cell (~21yrs)
Old NK cell (~70yrs)
Q1 2.38
Q2 0.82
Q4 1.31
Q3 95.5
Q1 0.057
Q2 0.96
Q4 0.85
Q3 98.1
CD3 - APC
CD56 - FITC
B
NK-Cell CD56
T-Cell CD3
p< 0.0001
p< 0.0001
Percentage of NK cell purity
Young NK Cells
Old NK Cells
N=6
C
Young Human Fibroblasts
Senescent Human Fibroblasts
SA-βGal staining
CPD:10
CPD: 60
D
p<0.001
Young HDF
Sen.HDF
SA-βGal+ cells/HPF
Young HDF
Sen. HDF
N=4
E
kDa
Yng HDF
Sen. HDF
21
p21
16
p16 INK4a
43
Actin
F
p<0.001
Young HDF
Senescent HDF
Expression of p21 and p16INK4a
p<0.001
p21
p16INK4a
N=4
G
Young MDF (50 days)
Old MDF (650 days)
SA-βGal staining
H
Young MDF (50 days)
Old MDF (650days)
0.0001
SA-βGal+ Cells/HPF
Young MDF
Old MDF
N=3
I
Young MDF
Old MDF
p16
Actin
J
Young MDF
Old MDF
p21
Actin
K
<0.0001
Young MDF
Old MDF
Expression of p16INK4a
Young MDF
Old MDF
N=3
L
0.0048
Young MDF
Old MDF
Expression of p21
Young MDF
Old MDF
N=3

Figure S1

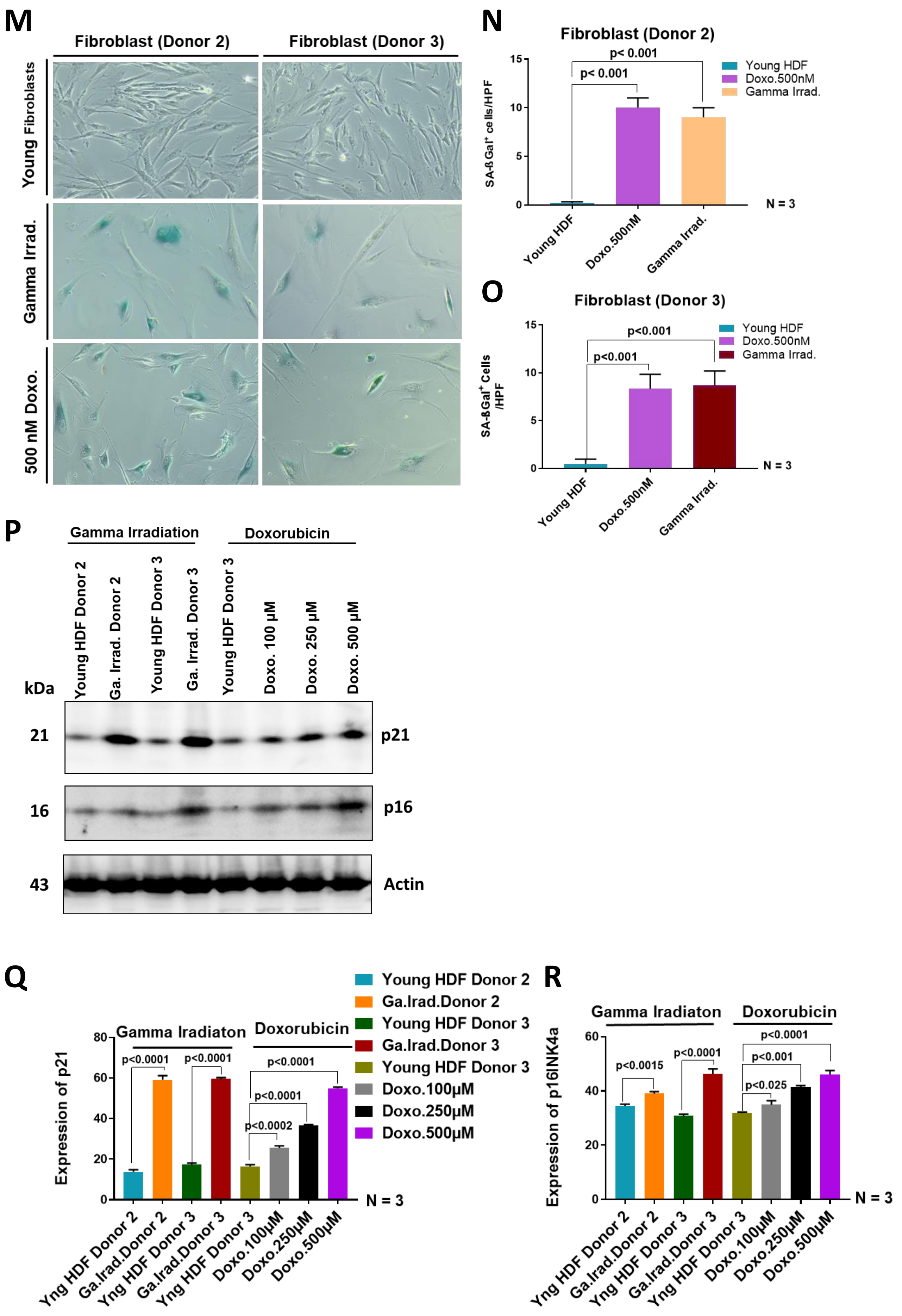

M
Fibroblast (Donor 2)
Fibroblast (Donor 3)
Young Fibroblasts
Gamma Irrad.
500 nM Doxo.
N
Fibroblast (Donor 2)
p< 0.001
p< 0.001
Young HDF
Doxo.500nM
Gamma Irrad.
N = 3
O
Fibroblast (Donor 3)
p<0.001
p<0.001
Young HDF
Doxo.500nM
Gamma Irrad.
N = 3
P
Gamma Irradiation
Doxorubicin
Young HDF Donor 2
Ga. Irrad. Donor 2
Young HDF Donor 3
Ga. Irrad. Donor 3
Young HDF Donor 3
Doxo. 100 μM
Doxo. 250 μM
Doxo. 500 μM
kDa
21
16
43
p21
p16
Actin
Q
Young HDF Donor 2
Ga.Irad.Donor 2
Young HDF Donor 3
Ga.Irad.Donor 3
Young HDF Donor 3
Doxo.100μM
Doxo.250μM
Doxo.500μM
Gamma Iradiaton
Doxorubicin
Expression of p21
p<0.0001
p<0.0001
p<0.0001
p<0.0001
p<0.0002
N = 3
Yng HDF Donor 2
Ga.Irad.Donor 2
Yng HDF Donor 3
Ga.Irad.Donor 3
Yng HDF Donor 3
Doxo.100μM
Doxo.250μM
Doxo.500μM
R
Gamma Iradiaton
Doxorubicin
Expression of p16INK4a
p<0.0015
p<0.0001
p<0.0001
p<0.001
p<0.025
N = 3
Yng HDF Donor 2
Ga.Irad.Donor 2
Yng HDF Donor 3
Ga.Irad.Donor 3
Yng HDF Donor 3
Doxo.100μM
Doxo.250μM
Doxo.500μM

Figure S2

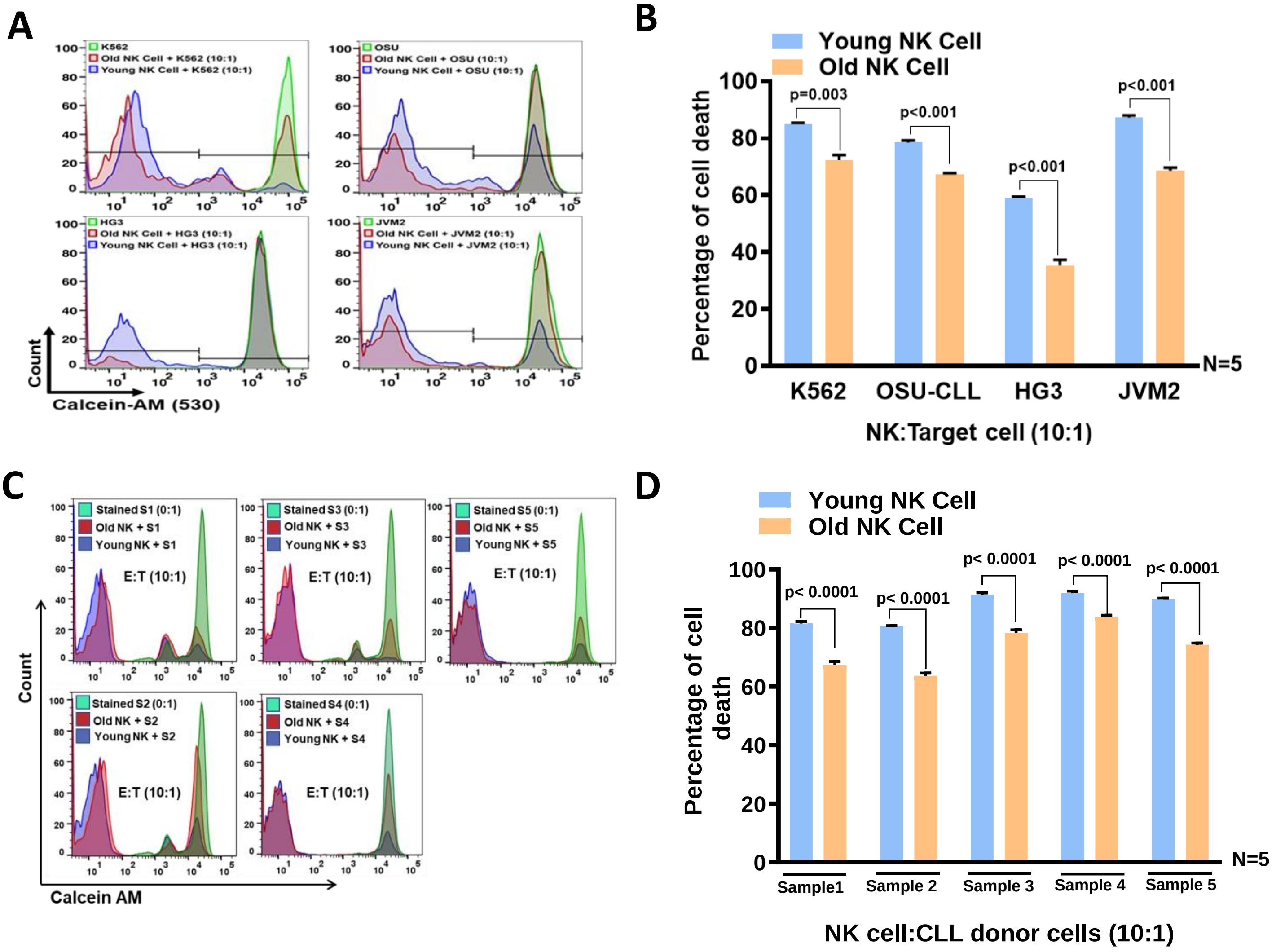
A
K562
Old NK Cell + K562 (10:1)
Young NK Cell + K562 (10:1)
OSU
Old NK Cell + OSU (10:1)
Young NK Cell + OSU (10:1)
HG3
Old NK Cell + HG3 (10:1)
Young NK Cell + HG3 (10:1)
JVM2
Old NK Cell + JVM2 (10:1)
Young NK Cell + JVM2 (10:1)
Count
Calcein-AM (530)
B
Young NK Cell
Old NK Cell
Percentage of cell death
p=0.003
p<0.001
p<0.001
p<0.001
N=5
K562
OSU-CLL
HG3
JVM2
NK:Target cell (10:1)
C
Stained S1 (0:1)
Old NK + S1
Young NK + S1
Stained S3 (0:1)
Old NK + S3
Young NK + S3
Stained S5 (0:1)
Old NK + S5
Young NK + S5
Stained S2 (0:1)
Old NK + S2
Young NK + S2
Stained S4 (0:1)
Old NK + S4
Young NK + S4
E:T (10:1)
Count
Calcein AM
D
Young NK Cell
Old NK Cell
Percentage of cell death
p< 0.0001
N=5
Sample1
Sample 2
Sample 3
Sample 4
Sample 5
NK cell:CLL donor cells (10:1)

Figure S3

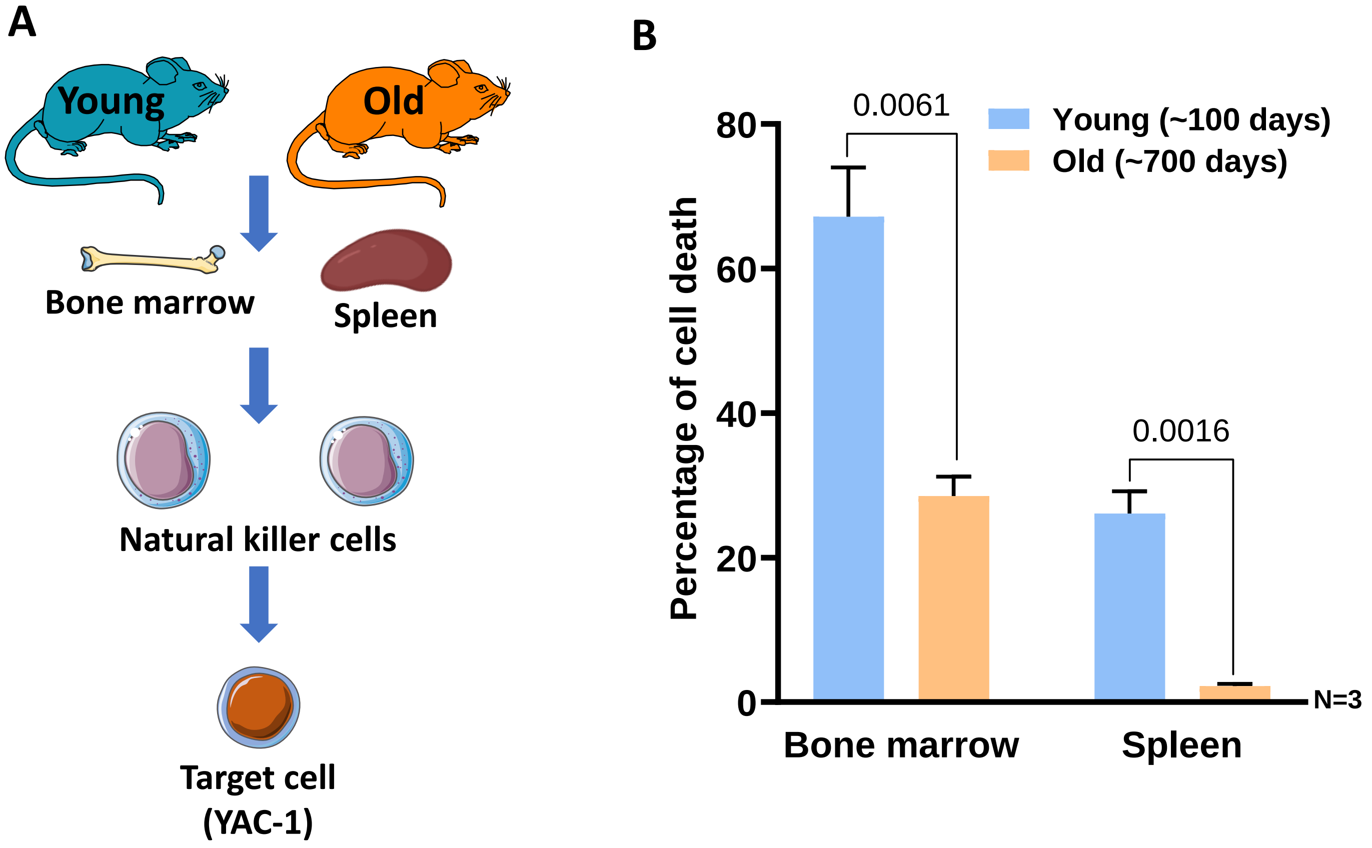
A
Young
Old
Bone marrow
Spleen
Natural killer cells
Target cell
(YAC-1)
B
Young (~100 days)
Old (~700 days)
0.0061
0.0016
Percentage of cell death
80
60
40
20
0
N=3
Bone marrow
Spleen

Figure S4

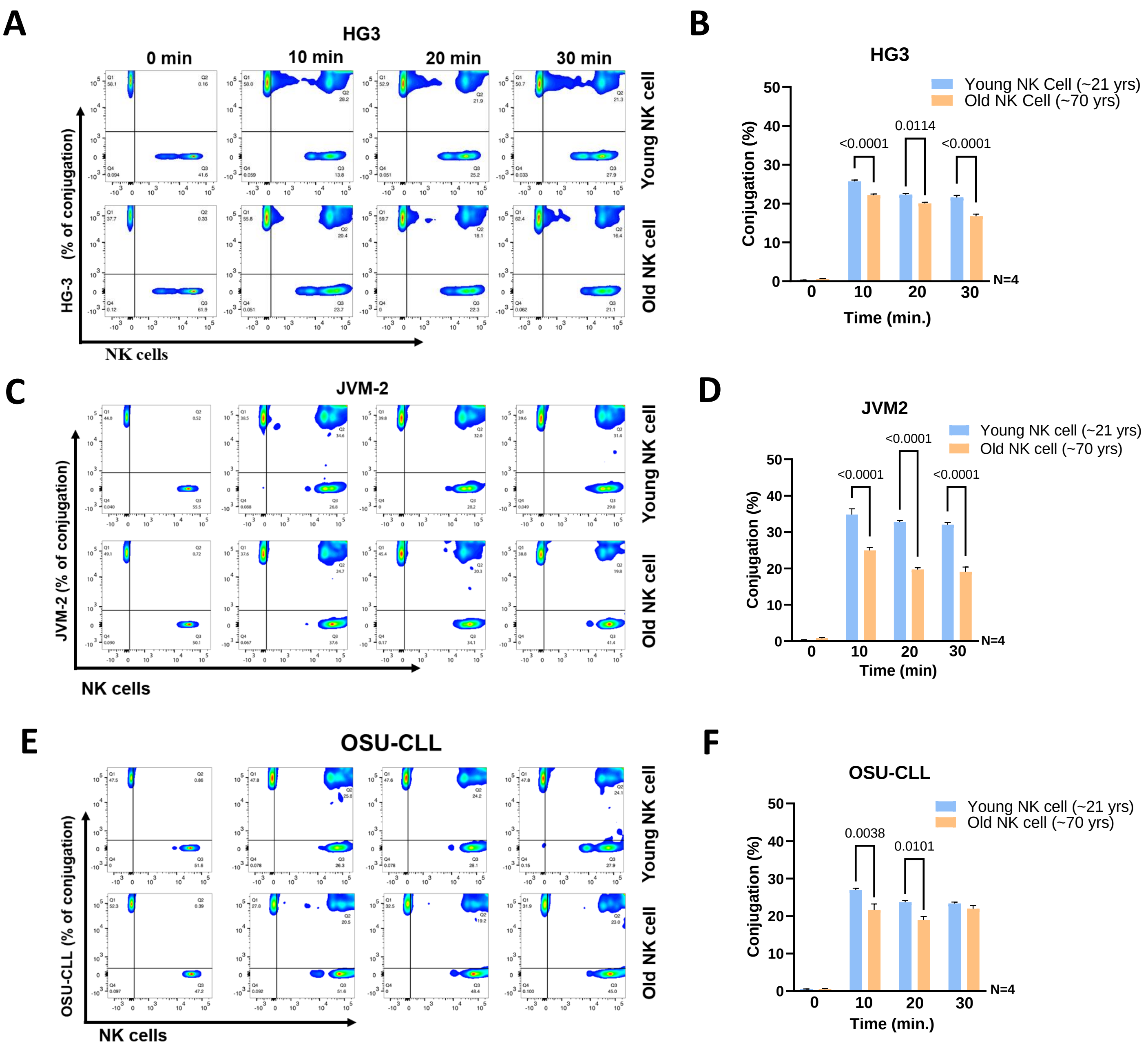
A
HG3
0 min
10 min
20 min
30 min
Young NK cell
Old NK cell
HG-3 (% of conjugation)
NK cells
B
HG3
Young NK Cell (~21 yrs)
Old NK Cell (~70 yrs)
Conjugation (%)
<0.0001
0.0114
<0.0001
N=4
Time (min.)
C
JVM-2
JVM-2 (% of conjugation)
Young NK cell
Old NK cell
NK cells
D
JVM2
Young NK cell (~21 yrs)
Old NK cell (~70 yrs)
Conjugation (%)
<0.0001
<0.0001
<0.0001
N=4
Time (min)
E
OSU-CLL
OSU-CLL (% of conjugation)
Young NK cell
Old NK cell
NK cells
F
OSU-CLL
Young NK cell (~21 yrs)
Old NK cell (~70 yrs)
Conjugation (%)
0.0038
0.0101
N=4
Time (min.)

Figure S5

A

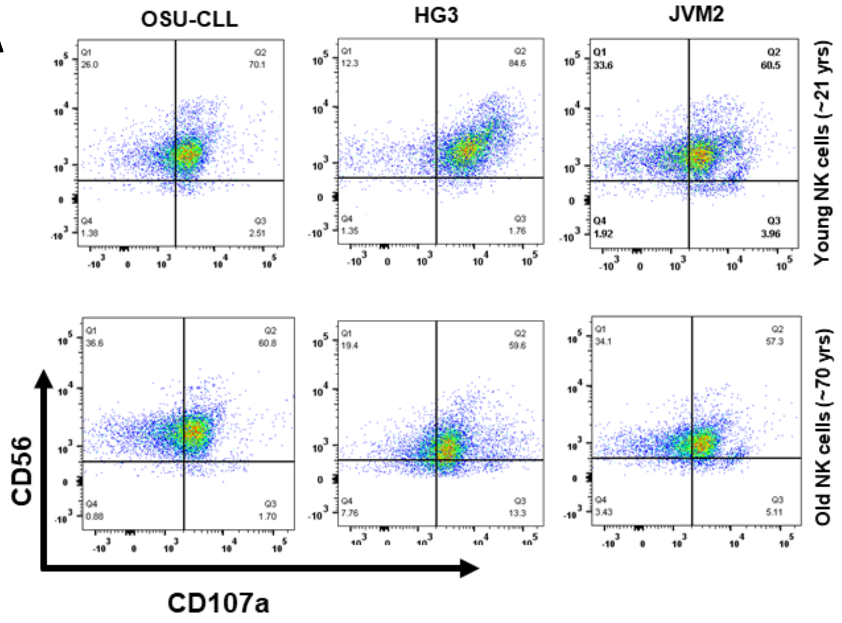

B

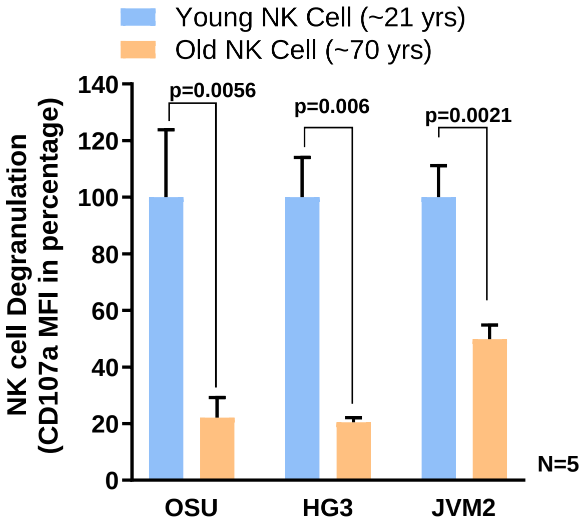

C

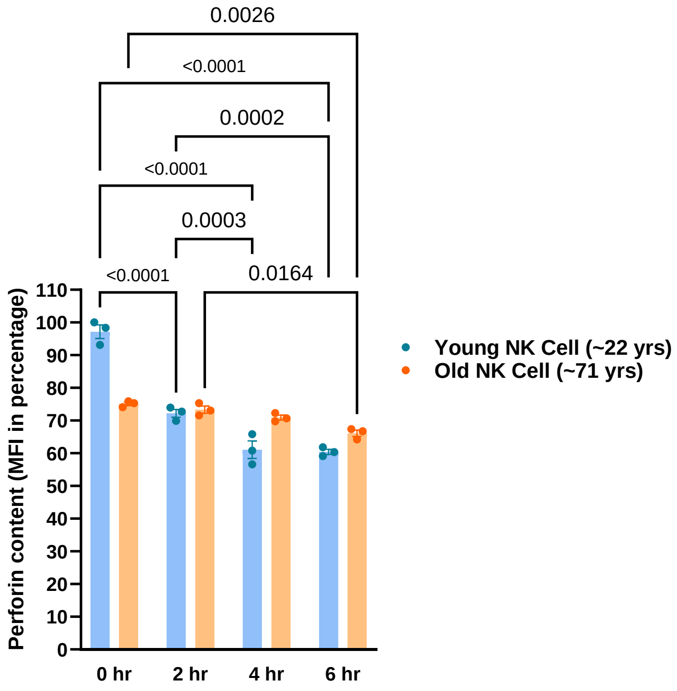

Figure S6

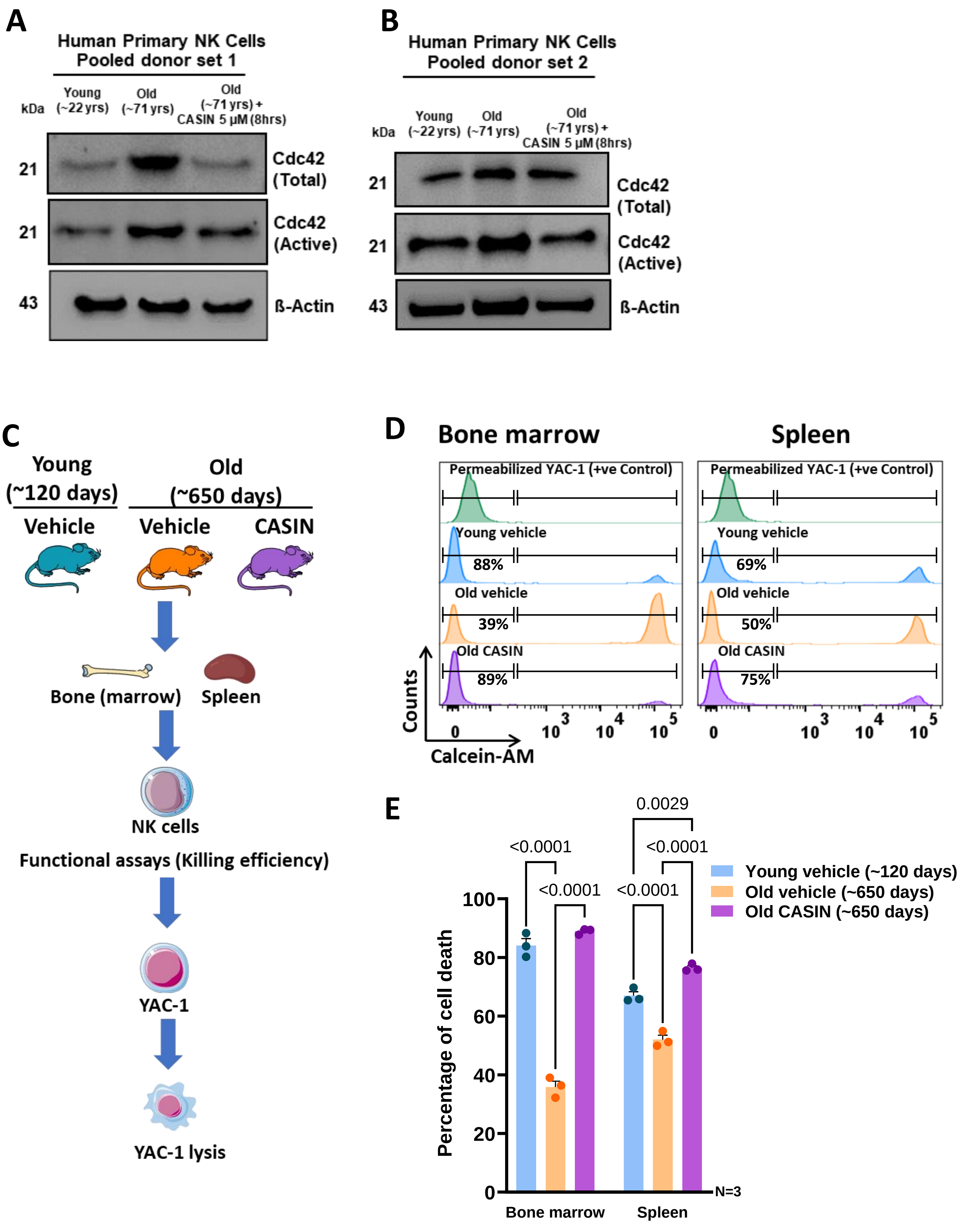

A
Human Primary NK Cells
Pooled donor set 1
kDa
Young (~22 yrs)
Old (~71 yrs)
Old (~71 yrs) + CASIN 5 µM (8hrs)
21
Cdc42 (Total)
21
Cdc42 (Active)
43
ß-Actin
B
Human Primary NK Cells
Pooled donor set 2
kDa
Young (~22 yrs)
Old (~71 yrs)
Old (~71 yrs) + CASIN 5 µM (8hrs)
21
Cdc42 (Total)
21
Cdc42 (Active)
43
ß-Actin
C
Young (~120 days)
Old (~650 days)
Vehicle
Vehicle
CASIN
Bone (marrow)
Spleen
NK cells
Functional assays (Killing efficiency)
YAC-1
YAC-1 lysis
D
Bone marrow
Spleen
Permeabilized YAC-1 (+ve Control)
Young vehicle
88%
69%
Old vehicle
39%
50%
Old CASIN
89%
75%
Counts
Calcein-AM
0
10^3
10^4
10^5
E
0.0029
<0.0001
<0.0001
<0.0001
<0.0001
Young vehicle (~120 days)
Old vehicle (~650 days)
Old CASIN (~650 days)
Percentage of cell death
0
20
40
60
80
100
N=3
Bone marrow
Spleen

Figure S7

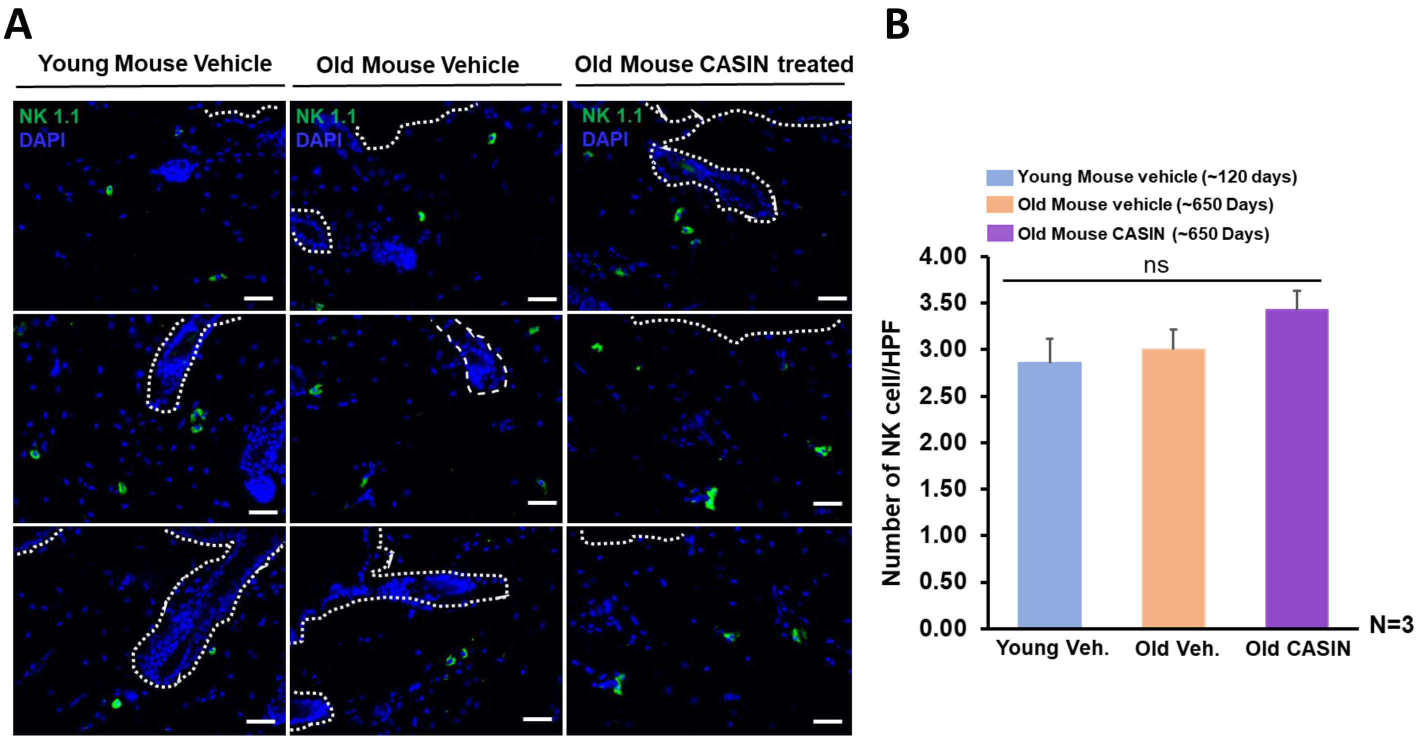
A
Young Mouse Vehicle
Old Mouse Vehicle
Old Mouse CASIN treated
NK 1.1
DAPI
B
Young Mouse vehicle (~120 days)
Old Mouse vehicle (~650 Days)
Old Mouse CASIN (~650 Days)
ns
Number of NK cell/HPF
4.00
3.50
3.00
2.50
2.00
1.50
1.00
0.50
0.00
Young Veh.
Old Veh.
Old CASIN
N=3
